# In- and Out-Group Effects on Social Perception and Empathy in Cocaine Use Disorder

**DOI:** 10.3389/fpsyt.2022.879016

**Published:** 2022-08-01

**Authors:** Tatjana Aue, Ann-Kathrin Kexel, Bruno Kluwe-Schiavon, Stephanie Bührer, Markus R. Baumgartner, Leila M. Soravia, Boris B. Quednow

**Affiliations:** ^1^Institute of Psychology, University of Bern, Bern, Switzerland; ^2^Experimental and Clinical Pharmacopsychology, Department of Psychiatry, Psychotherapy and Psychosomatics, Psychiatric University Hospital Zurich, University of Zurich, Zürich, Switzerland; ^3^Center for Forensic Hair Analytics, Institute of Forensic Medicine, University of Zurich, Zürich, Switzerland; ^4^Translational Research Center, University Hospital of Psychiatry, University of Bern, Bern, Switzerland; ^5^Neuroscience Center Zurich, Swiss Federal Institute of Technology Zurich and University of Zurich, Zürich, Switzerland

**Keywords:** stimulants, cocaine, social cognition, empathy, social perception, optimism bias

## Abstract

Earlier research revealed that cocaine users display impairments in emotional but not necessarily in cognitive empathy. However, no study to date has tested whether empathy is generally altered or whether impairments are restricted to specific social targets. The current investigation addresses this open question. In addition, we examined whether attributions of warmth and competence as well as personal future expectancies differed between cocaine users and substance-naïve controls. Twenty-two chronic cocaine users and 40 stimulant-naïve controls specified their perceived warmth and competence for four social targets [in-group member, opposite consumption out-group member (cocaine user for controls and non-user for cocaine user), opposite consumption out-group member of opposite gender, and elderly person]. They also specified their cognitive and emotional empathy for these four targets facing eight desirable and eight undesirable events. Finally, they rated the likelihood of these scenarios happening to themselves. Both cocaine users and controls attributed lower warmth to cocaine-using than non-using targets. Comparably, no in-group preference was observed in cocaine user’s emotional empathy ratings, and greater denigration of the in-group was associated with higher frequency and doses of cocaine consumption. In addition, cocaine users rated both desirable and undesirable events as more likely to happen to themselves than did controls. Results show that substance-naïve individuals stigmatize cocaine users. They further point to compromised self-esteem in cocaine users resulting from such stigmatization. Interventions should address stigmatization processes to break the vicious circle of mutual social distancing and stronger dedication to the drug.

“Happiness lies within one’s self, and the way to dig it out is cocaine.”

  — Aleister Crowley, Diary of a Drug Fiend

## Introduction

Recent findings suggest that cocaine users orient toward the drug in order to augment experiences of reward – experiences that they do not (or no longer) obtain from social interactions with others (1, 2). The inability to attain social rewards may be intimately linked with deficient social capacities [e.g., impaired empathic responding; (3, 4)] and diminished sensitivity to social rewards (2, 5, 6). Accordingly, it has been suggested that devotion to cocaine and withdrawal from friends and family mutually influence each other, resulting in a vicious circle (7). Notably, both recreational and dependent cocaine consumers have smaller social networks (8) and display various particularities in social cognition and interactions, including diminished cooperativeness and compliance with social norms (9), lower emotional empathy and disturbed perspective taking (8), a stronger focus on efficiency than fairness in money distribution games (10), and impaired joint attention (5). Consequently, to better understand the involvement of cocaine in social functionality and addiction, investigating social deficits in chronic cocaine use is warranted.

The present investigation focuses on associations between perceptions of warmth and competence in others, and empathic responding, on the one hand, and cocaine use, on the other. Social deficits and conflicts may arise due to altered perceptions of others. For instance, the Stereotype Content Model (11, 12) states that social targets are classified along two orthogonal dimensions: warmth and competence, leading to different affective experiences, attitudes, and behaviors in an observer (11–16). Correspondingly, the current study examined whether cocaine users display particularities in attributions of warmth and competence to different social targets.

Altered processing related person perceptions might also explain the above-reported particularities in empathic responding that accompany cocaine use [cf. Aue et al. (17)]. Contemporary accounts of empathy distinguish between two major concepts, namely cognitive and emotional empathy (18–20). Cognitive empathy requires mentalizing and relates to an individual’s capacity to infer other people’s mental states, thereby ensuring understanding of other people’s feelings. By contrast, emotional empathy describes the appropriation of the affective feeling state of a social target by the perceiver and, hence, involves affective sharing.

Some earlier studies associated cocaine use with impaired cognitive empathy, specifically with reduced emotional intelligence (21) and emotion recognition from faces (22). Another recent comprehensive investigation in a large-scale sample revealed cognitive empathy in cocaine users to be little affected (8, 23), with impairments being limited to auditory stimuli (i.e., recognition of emotions in the voice) or multisensory integration [i.e., regarding (mis)matching information in faces and voices]. There was further evidence that deficient cognitive empathy was restricted to severe cocaine consumption (i.e., addiction) combined with attention-deficit/hyperactivity disorder (ADHD) symptomatology (8, 24), with only long-term users overinterpreting social signs and attributing exaggerated emotions to others. By contrast, in the same large-scale investigation (8, 23), recreational and dependent cocaine users demonstrated marked impairments in both implicit and explicit assessments of emotional empathy, and the degree of impairment was positively correlated with lifetime extent (related to both dose and duration) of cocaine consumption and negatively correlated with social network size. Interestingly, longitudinal data suggest that emotional empathy and prosocial behavior may recover when cocaine use is strongly reduced or quitted (4). In sum, research on empathy related to cocaine use reveals weak links with cognitive empathy deficits, but strong associations with emotional empathy deficits. While suggestive, these observations ask for further refinement. For instance, it is still unclear, whether cocaine users’ empathic responses discriminate between different social targets.

Previous studies in the general population have consistently revealed that individuals display greater (mostly emotional) empathy for in-group (i.e., people they identify with) compared with out-group members (25–28). Moreover, people’s empathic responding clearly differentiates between different kinds of social out-groups (17). Because chronic cocaine use has been linked to social isolation and diminished sensitivity to social rewards, it is possible that users perceive the in-group and out-groups differently than do non-users, which then feeds back to their empathic responses. Accordingly, in the current study, we examined differences between cocaine users and non-using controls regarding (a) perceptions of warmth, (b) perceptions of competence, (c) cognitive empathy, and (d) emotional empathy displayed for/toward an in-group member and three different kinds of out-group members (specified below).

Furthermore, apart from showing altered processing of social stimuli, cocaine users reveal particularities in processes that relate to the self. Among others, acute effects of cocaine have been reported to subsume euphoria, augmented ego distinctiveness, exaggerated self-confidence, as well as an excessive sense of mastery over fate (29) – with regulation problems related to ego functions and reality testing arising as negative aftereffects once the acute effects of the drug have faded out (also known as “crash”). Moreover, chronic use of cocaine has been reported to go along with feelings of depression or emotional blunting (30–32). Together, therefore, these observations point to deviated future outlooks in cocaine users. Accordingly, we broach the idea that cocaine use relates to future expectancies. The majority of people in our population expects their personal future to more likely provide positive rather than negative outcomes (33, 34). What is more, they also believe that desirable (undesirable) events are more (less) likely to happen to themselves than to a comparison person of same age and gender (35). Cocaine use may particularly predispose to such thinking, with the negative postacute effects of the drug possibly shifting the bias into the opposite direction (i.e., into a pessimism bias with an overestimation of undesirable over desirable future outcomes).

Importantly, there are other facts that made us hypothesize that cocaine use and optimistically biased expectancies cohere. Reward impulsivity and wanting (36) have been put forth as key factors in research on cocaine use (10, 37), and these same factors are considered essential in theories on optimism bias [see Kress and Aue (34)]. Furthermore, structural and metabolic aspects of some important brain regions (e.g., inferior frontal gyrus, medial prefrontal cortex, anterior cingulate cortex, and striatum) involved in optimism and optimism bias (33, 34) have been shown to be affected by cocaine use (37–41). Consequently, we tested whether cocaine users are characterized by peculiarities in optimistic outlooks.

In the current investigation, chronic cocaine users as well as stimulant-naïve healthy control participants imagined different desirable and undesirable scenarios and specified their cognitive and emotional empathy toward four different social targets experiencing those scenarios: one in-group member (cocaine user of same gender for cocaine using participants; non-cocaine user of same gender for control participants) and three different out-group members (for cocaine users: an elderly person of same gender, a same-aged non-cocaine using target of same gender, and a same-aged non-cocaine using target of the opposite gender; for control participants: an elderly person of same gender, a same-aged cocaine-dependent target of same gender, and a same-aged cocaine-dependent target of the opposite gender). They further designated their level of identification with each social target (manipulation check to verify that greatest identification arose with respect to the presumed in-group) and how warm and competent they perceived these targets. Our participants also specified their personal likelihood of experiencing any of the scenarios involved in the social task (assessment of self-related expectancies, targeting optimism bias). Based on the literature reviewed, we tested the following hypotheses (summarized in Table 1):

**TABLE 1:** List of hypotheses.

Index	Dependent variable	Hypothesis	Specification of results/additional comments
**H1a**	**Perceived warmth** Controls	Cocaine-using targets are rated as less warm than in-group and elderly out-group^a^	Higher warmth attributions in females compared with males
**H1b**	**Perceived competence** Controls	Highest competence attributed to in-group, then elderly, then cocaine-using targets^b^	
**H1c**	**Perceived warmth** Comparison cocaine users vs. controls	Lower ratings of warmth in cocaine users than controls (esp. for in-group)^b^	Cocaine-consuming social targets are perceived as colder than non-cocaine-consuming targets by both cocaine users and controls: lower warmth ratings for in-group in cocaine users than controls; cocaine users rate non-consuming targets as warmer than controls rate cocaine-consuming targets
**H1d**	**Perceived competence** Comparison cocaine users vs. controls	Lower ratings of competence in cocaine users than controls (esp. for in-group)^b^	
**H1e**	**Perceived warmth** Cocaine users	Little or no differentiation between the different social targets^b^	Higher warmth attributions in females compared with males
**H1f**	**Perceived competence** Cocaine users	Little or no differentiation between the different social targets^a^	
**H2a**	**Cognitive empathy** Controls	Positive scenarios: greater cognitive empathy for elderly and in-group targets than for cocaine-using targets^a/b^; Negative scenarios: no difference between the social targets^a^	Positive scenarios: greater cognitive empathy expressed for elderly than cocaine-using targets; no difference of either with respect to in-group character
**H2b**	**Cognitive empathy** Comparison cocaine users vs. controls	Comparable overall levels of cognitive empathy in cocaine users and controls^a^	
**H2c**	**Cognitive empathy** Cocaine users	Little or no differentiation between the different social targets^b^	No reduced differentiation compared with controls, but no differentiation between positive and negative scenarios; attribution of stronger feelings to the three out-group characters compared with the in-group character; greater cognitive empathy in female than male cocaine users
**H3a**	**Emotional empathy** Controls	Highest emotional empathy expressed for elderly, then in-group, then cocaine-using targets^a^	Difference between elderly and in-group is not statistically significant
**H3b**	**Emotional empathy** Comparison cocaine users vs. controls	Lower ratings of emotional empathy in cocaine users than controls^b^	
**H3c**	**Emotional empathy** Cocaine users	Little or no differentiation between the different social targets^a^	
**H4a**	**Self-related future expectancies** Controls	Higher likelihood ratings for positive compared with negative scenarios (optimism bias)^a^	
**H4b**	**Self-related future expectancies** Comparison cocaine users vs. controls	Altered optimism bias in cocaine users (no directed hypothesis)^b^	Cocaine users attribute greater likelihood to both positive and negative scenarios than do controls

*^a^Study data are supportive of hypothesis.*

*^b^Study data are not supportive of hypothesis.*

### Perception of the Social Targets

Earlier research [e.g., Aue et al. (17), Dricu et al. (42, 43), Moser et al. (44)] has shown that substance-naïve participants stigmatize substance users in that they attribute low warmth and low competence to them. By contrast, the same individuals perceive in-group members as both warm and competent and elderly persons as warm but little competent. Accordingly, we predicted controls to rate the in-group and the elderly out-group as warmer than both cocaine-dependent social targets (H1a). In addition, cocaine targets were expected to be rated as less competent than the remaining social targets by the control participants, with the elderly population lying in between the cocaine targets and the in-group target (H1b). Because of the reported link between cocaine use and social isolation, we further predicted that cocaine users would see others in less bright colors, thereby attributing lower warmth to the different social targets than controls (the difference being particularly pronounced for the in-group target; H1c). The same hypothesis was tested for the competence ratings (H1d). Finally, due to greater social distancing from others, cocaine users were hypothesized to demonstrate comparably small differences in their warmth and competence ratings for the different social targets (H1e and H1f).

### Cognitive Empathy

Consistent with earlier observations (17), we predicted controls to display greater cognitive empathy for the elderly and in-group targets than the cocaine-using targets – but solely for positive scenarios (no difference for negative scenarios because of society’s conviction that everybody has the right to feel bad; H2a). Based on the finding that cognitive empathy is virtually unaffected by cocaine use (8, 23), we expected controls and cocaine users to display comparable overall levels of cognitive empathy (H2b). Yet, we predicted our cocaine-using participants to display less differentiation between the different social targets (H2c).

### Emotional Empathy

In line with previous findings (17), we expected control participants to state the highest emotional empathy for the elderly target, the lowest for the cocaine-using targets, and the in-group placed in between (H3a). Based on the demonstrated impairments in emotional empathy in cocaine use (8, 23), we further predicted cocaine users to display overall lower levels of emotional empathy than control participants (H3b). Finally, cocaine users were hypothesized to reveal reduced differentiation between the social targets (H3c).

### Self-Related Future Expectancies

Control participants were predicted to display an optimism bias (33), with higher likelihood ratings for positive compared with negative events anticipated for their personal future (H4a). This bias was hypothesized to be altered in cocaine users (H4b; no directed hypothesis).

## Materials and Methods

### Participants

The present sample is a subsample of a previously published study (32). Whereas all *n* = 40 stimulant-naïve healthy control participants had completed the tasks of interest for the current investigation, time limitations resulted in only *n* = 22 (out of 59) cocaine users doing so. Cocaine users were included in the study if cocaine was the primary illegal drug they used, if a lifetime cumulative consumption of at least 100 g of cocaine was estimated by self-report, and if their current abstinence duration was <6 months. General exclusion criteria comprised a family history of genetically mediated psychiatric disorders (*h*^2^ > 0.5, e.g., autism, schizophrenia, and bipolar disorder), any severe neurological disorder or brain injury, intake of medication with potential action at the central nervous system during the last 7 days, and participation in a large previous study from our lab, the Zurich Cocaine Cognition Study (8).

Controls were excluded if they had Axis I adult psychiatric disorders according to the Diagnostic and Statistical Manual of Mental Disorders-IV – Text Revision [DSM-IV-R; (45)] or recurrent illegal substance use (>15 occasions lifetime, with the exception of cannabis for reasons of participant matching). We excluded cocaine users with regular use of illegal substances other than cocaine, such as heroin or other opioids (with the exception of cannabis), a polysubstance use pattern, and an Axis I adult psychiatric disorder diagnosis (e.g., schizophrenia, bipolar disorder, current major depressive episode, eating disorders, and current anxiety disorder) according to DSM-IV, with exception for cocaine, cannabis, and alcohol abuse/dependence, previous depressive episodes, and ADHD.

Experimental protocols, methods of data collection, data handling, and data analysis were approved by the Ethics Committee of the Canton Zurich (BASEC ID 2016-00278) and are fully in accord with the Declaration of Helsinki (46).

### Experimental Tasks

**FIGURE 1:**
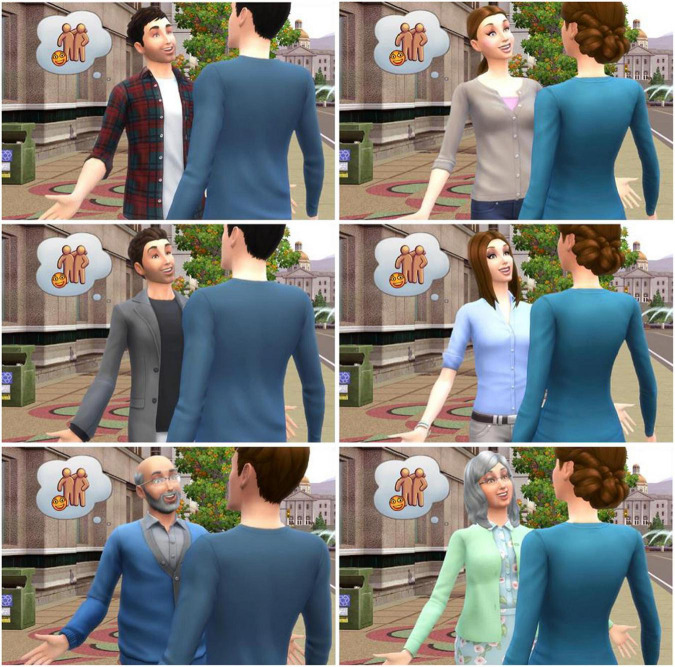
Social targets included in the current study. From top to bottom: non-cocaine user, cocaine user, elderly person. Left, male; right, female.

Included tasks were programmed with E-Prime 2.0 Professional (version 2.0.10.356; Psychology Software Tools, Pittsburgh, PA, United States). All but one task (self-related expectancies) comprised four social targets, displayed as still animations of an in-group member and three out-group members (Figure 1). Specifically, for cocaine participants/non-cocaine participants, the included characters were as follows (a) a cocaine user/non-cocaine user of same gender as the participant (in-group; IG), (b) a non-cocaine user/cocaine user of same gender (out-group use; OG_u_), (c) a non-cocaine user/cocaine user of different gender (i.e., double out-group termed out-group use + gender; OG_ug_), and (d) an elderly person of same gender (out-group age; OG_a_). Social targets (and backgrounds of the different scenarios, relevant for the empathy ratings only) were created with *The Sims 4* (Electronic Arts, CA, United States). All stimuli were controlled in brightness and contrast using MATLAB R2017a (The Math Works, Inc., MA, United States).

#### Level of Identification With the Different Social Targets (Manipulation Check)

Our participants rated their similarity with each of the four social targets on the Inclusion of Other in the Self [IOS; (47)] scale (Supplementary Figure 1). The IOS scale consists of seven pairs of circles that vary in their degree of overlap, describing the perceived similarity of a social target with the self. Possible scores ranged from 1 (very dissimilar, almost no overlap) to 7 (very similar, almost complete overlap).

#### Perceived Warmth and Perceived Competence of the Different Social Targets

Participants stated the perceived warmth for each of the four social targets on a continuous visual analog scale with the endpoints “not at all warm” (yielding a stored value of 0) and “very warm” (resulting in a stored value of 100; Supplementary Figure 2). In addition, they rated each social target’s perceived level of competence on a scale with the endpoints “not at all competent” (yielding a stored value of 0) and “very competent” (resulting in a stored value of 100).

#### Cognitive and Emotional Empathy for the Different Social Targets

Cognitive empathy relates to metacognitive abilities and was assessed *via* the question: “In your opinion, how good/bad does the depicted character feel in this specific situation?” Emotional empathy refers to affective sharing and was assessed *via* the question: “How good/bad do you feel when you see the depicted character in this specific situation?” Cognitive and emotional empathy ratings for the four social targets were given on continuous visual analog scales that ranged from −50 (feeling “very bad”) to 50 (feeling “very good”; Supplementary Figure 3). For each social target, participants rated eight positive and eight negative scenarios [matched with respect to event frequency and controllability as assessed in an earlier study (42); refer to Supplementary Material for the exhaustive list of events]. Thus, participants specified each their cognitive and their emotional empathy for 64 scenario × target combinations.

#### Self-Related Future Expectancies (Optimism Bias)

Our participants rated their personal likelihood (scale range: 0–100%; corresponding to “not at all” to “absolutely certain”) of encountering each of eight negative and eight positive future scenarios (identical to the scenarios included in the empathy task) on a continuous visual analog scale.

### Clinical and Substance-Related Assessment

The psychopathological assessment was carried out with the Structured Clinical Interview I [SCID-I; (48)] according to DSM-IV-R (45) to determine the presence of DSM-IV Axis I psychiatric disorders. The Structured Clinical Interview for DSM-IV Axis II Disorders questionnaire [SCID-II; (49)] was used to assess cluster B personality disorder symptoms. The German vocabulary test Mehrfachwahl-Wortschatz-Intelligenztest [MWT-B; (50)] was applied to estimate premorbid verbal intelligence. ADHD symptoms were collected with the ADHD self-rating scale [ADHD-SR; (51)]. Depressive symptomatology was measured with the German version of the Beck Depression Inventory (52). For the determination of the social network size, an adapted version of the Social Network Questionnaire [SNQ; (8, 53)] was administered. Self-reported drug use was assessed with the structured and standardized Interview for Psychotropic Drug Consumption (54).

### Urine and Hair Toxicological Analysis

For the drug urine screening a semi-quantitative enzyme multiplied immunoassay method was used (Dimension RXL Max, Siemens, Erlangen, Germany). In addition, quantitative analysis of hair samples using liquid chromatography tandem mass spectrometry (LC-MS/MS) was applied to investigate substance use over the last 4 months as represented in the proximal 4 cm-segment of the hair samples. In total 88 compounds were assessed [for details see Scholz et al. (55)].

### Procedure

Upon their arrival at the laboratory, participants signed an informed consent form. Subsequently, they underwent a sequence of tasks, interviews, sampling of urine and hair, and psychometric instruments [see Kluwe-Schiavon et al. (32)], of which only the relevant ones are outlined here. Specifically, participants rated in a fixed sequence (1) their personal likelihood of encountering different positive and negative future events; (2) their cognitive and emotional empathy for four different social targets experiencing the same set of events; (3) how warm and competent they perceived the four different targets to be; and (4) how similar they felt to the social targets. After the completion of the tasks, participants were debriefed.

### Data Preparation and Analysis

#### Level of Identification With (Manipulation Check), Perceived Warmth of and Perceived Competence of the Different Social Targets

For each dependent variable, we conducted a repeated-measures analysis of variance (ANOVA) with the between-participants factors *Group* (cocaine and control) and *Gender* (male and female) and the within-participants factor *Target Character* (IG, OG_u_, OG_ug_, and OG_a_).

#### Cognitive and Emotional Empathy for the Different Social Targets

Because our data should reflect the appropriateness of attributed (cognitive empathy) or experienced (emotional empathy) affective states, we reversed the scores given for the undesirable scenarios, so that higher scores represent greater (assigned) suffering. For the desirable scenarios, such recoding was not indicated (higher scores already reflect more positive affect). For each participant, an average cognitive empathy score and an average emotional empathy score were calculated for each combination of *Scenario Valence* (negative and positive) and *Target Character* (IG, OG_u_, OG_ug_, and OG_a_). Two repeated-measures ANOVAs with the between-participants factors *Group* (cocaine and control) and *Gender* (male and female) and the within-participants factors *Scenario Valence* (negative and positive) and *Target Character* (IG, OG_u_, OG_ug_, and OG_a_) were calculated – one for the participants’ cognitive, and another for the participants’ emotional empathy ratings.

#### Self-Related Future Expectancies

Rating scores were averaged for each level of scenario valence in every participant. A repeated-measures ANOVA with the between-participants factors *Group* (cocaine and control) and *Gender* (male and female) and the within-participants factor *Scenario Valence* (negative and positive) was calculated on the averaged likelihood ratings.

#### All Dependent Variables

Because we only had six female cocaine-consuming participants, we did not include any term relating to the interaction between the factors *Group* and *Gender* in our ANOVAs.

## Results

Pearson product moment correlation coefficients for the association between the different social constructs are displayed in Supplementary Material. To enable better comprehension of the results presented, only significant and meaningful non-significant effects are described. A complete overview of effects can be found in Supplementary Material.

### Characterization of the Study Sample

The final sample included in our analyses consisted of 62 participants, 22 chronic cocaine users (16 male) and 40 controls (24 male). Age ranged between 21 and 51 years (*M* = 30.4 years, SD = 6.57 years). Table 2 summarizes the participants’ age, verbal intelligence, and characteristics assessed *via* clinical scales. It also displays substance use features. Cocaine users were characterized by lower verbal intelligence, higher depression and antisociality scores, as well as higher scores on the ADHD-SR than were controls. Compared with controls, cocaine users also reported greater weekly alcohol use. Finally, self-reported substance use and hair toxicological results of cocaine users showed a clear preference for cocaine over other substances.

**TABLE 2:** Overview of participants’ age, verbal intelligence, scores on clinical scales, and consumption features.

Comparison of	Cocaine users	Controls			
**Measure**	***M* (SD)**	***M* (SD)**	**Test statistic**	**df**	***p***
Age, years	32.3 (6.0)	29.3 (6.7)	*t* = 1.75^a^	60	0.086
Gender (m/f)^f^	16/6 (73/27)	24/16 (60/40)	χ^2^ = 1.00^b^	1	0.316
Verbal IQ	95.9 (7.0)	102.2 (8.6)	*t* = −2.93^a^	60	0.005
ADHD-SR, score	16.4 (11.0)	9.7 (9.7)	*t* = 2.46^a^	60	0.017
BDI, score	7.5 (6.7)	3.5 (5.2)	*t* = 2.57^a^	57^e^	0.013
SCID-II histrionic, score	1.9 (1.4)	1.6 (1.4)	*t* = 0.65^a^	60	0.517
SCID-II narcissistic, score	3.9 (2.8)	2.8 (2.5)	*t* = 1.62^a^	60	0.111
SCID-II borderline, score	4.6 (3.2)	2.8 (2.9)	*t* = 2.30^a^	60	0.025
SCID-II antisocial, score	5.9 (23.1)	2.7 (2.4)	*t* = 4.28^a^	60	<0.001
SNQ (network size), score	12.7 (8.8)	18.8 (14.9)	*t* = 1.74^a^	60	0.087
**Substance use features**					
***Nicotine***					
Smoker^f, h^	20 (91)	34 (85)	χ^2^ = 0.54^b^	1	0.462
Cigarettes per week^g, i, j^	78.8 (10.00–245.00)	70.0 (7.0–175.0)	*U* = 278.00^c^		0.264
***Alcohol***					
Pure alcohol, grams per week^g, j^	145.5 (35.5–1,415.1)	75.1 (0.2–375.7)	*U* = 268.50^c^		0.012
***Cannabis***					
Years of use	10.8 (8.8)	6.3 (6.2)	*t* = 2.12^d^	32.7	0.042
Times per week^g, j^	0.0 (0.0–3.0)	0.0 (0.0–2.0)	*U* = 359.50^c^		0.191
Grams per week^g, j^	0.0 (0.0–0.5)	0.0 (0.0–1.2)	*U* = 349.50^c^		0.152
Cumulative lifetime grams^g^	692 (0.0–25,719)	3.6 (0.0–2,630)	*U* = 232.50^c^		0.002
Urine toxicology (pos)^f^	4 (18)	2 (5)	χ^2^ = 2.82^b^	1	0.093
***Cocaine***					
Times per week^j^	2.3 (2.4)				
Grams per week^j^	4.0 (7.4)				
Abstinence (days)	19.0 (32.7)				
Cumulative lifetime grams	1,552.0 (1,485.0)				
Cocaine_total_, pg/mg in hair^k^	31,240 (51,230)				
Cocaine, pg/mg in hair	21,469 (38,557)				
Benzoylecgonine, pg/mg in hair	9,239 (14,302)				
Norcocaine, pg/mg in hair	533 (800)				
Cocaethylene, pg/mg in hair	697 (1,130)				
Urine toxicology (pos)^f^	6 (27)	0 (0)	χ^2^ = 12.10^b^	1	<0.001
DSM-IV cocaine dependency (lifetime)^f^	19 (86)	0 (0)	n/a	n/a	n/a
DSM-IV cocaine abuse (lifetime)^f^	21 (96)	0 (0)	n/a	n/a	n/a

*^a^Independent t-test.*

*^b^χ^2^ test for frequency data.*

*^c^Mann–Whitney U test.*

*^d^Welch’s t-test.*

*^e^Due to missing scores from 3 control participants.*

*^f^n (%) is reported.*

*^g^Median (range) is reported.*

*^h^Individuals were considered smokers if they smoked ≥7 cigarettes/week.*

*^i^Only for smokers.*

*^j^Average use during the last 6 months.*

*^k^Cocaine_total_ (= cocaine + benzoylecgonine + norcocaine) as a more robust parameter (74). Verbal IQ, verbal intelligence quotient estimated with the German vocabulary test (MWT-B); ADHD-SR, ADHD self rating scale; BDI, Beck Depression Inventory; SCID-II, Structured Clinical Interview for DSM-IV Axis II Disorders questionnaire; SNQ, Social Network Questionnaire.*

### Level of Identification With the Different Social Targets (Manipulation Check)

**FIGURE 2:**
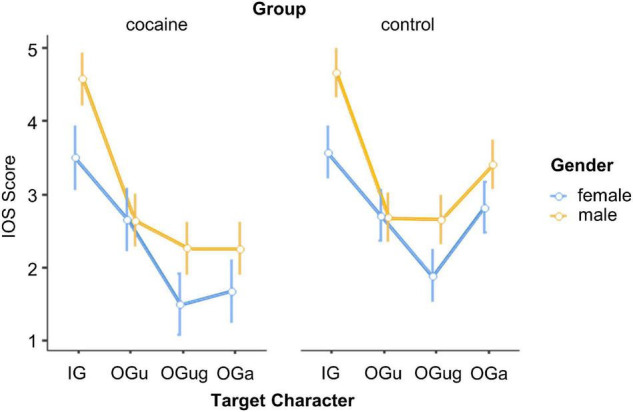
Identification with the social targets. IOS, Inclusion of Other in the Self Scale; IG, in-group; OG_u_, out-group use; OG_ug_, out-group use + gender; OG_a_, out-group age. Error bars, standard errors.

A repeated-measures ANOVA with the between-participants factors *Group* (cocaine and control) and *Gender* (male and female), and the within-participants factor *Target Character* (IG, OG_u_, OG_ug_, and OG_a_) was calculated on the similarity ratings. The main effect of *Target Character* achieved significance, *F*(3,177) = 17.74, *p* < 0.001, $\eta_{p}^{2}=0.23$ (cf. Figure 2). As intended, our participants identified more strongly with their pre-determined in-group (*M* = 4.1, LCI = 3.7, UCI = 4.5) compared with the three out-groups (OG_u_: *M* = 2.7, LCI = 2.3, UCI = 3.1; OG_ug_: *M* = 2.1, LCI = 1.7, UCI = 2.5; OG_a_: *M* = 2.5, LCI = 2.1, UCI = 3.0; pairwise Tukey HSD comparisons with the IG: all *p*-values < 0.001; remaining *p*-values > 0.169). In addition, there was a significant main effect of *Gender*, *F*(1,59) = 6.47, *p* = 0.014, $\eta_{p}^{2}=0.10$, with males more strongly identifying with the targets than females (*M*s = 3.0 and 2.4, respectively).

### Perceived Warmth of the Different Social Targets

A repeated-measures ANOVA with the between-participants factors *Group* (cocaine and control) and *Gender* (male and female) and the within-participants factor *Target Character* (IG, OG_u_, OG_ug_, and OG_a_) was run on our participants’ warmth ratings. Not supportive of our H1c, the main effect of *Group* was not significant, *F*(1,59) = 0.29, *p* = 0.590, $\eta_{p}^{2}=0.01$, suggesting that the two groups did not differ in the overall amount of warmth attributed to the social targets.

**FIGURE 3:**
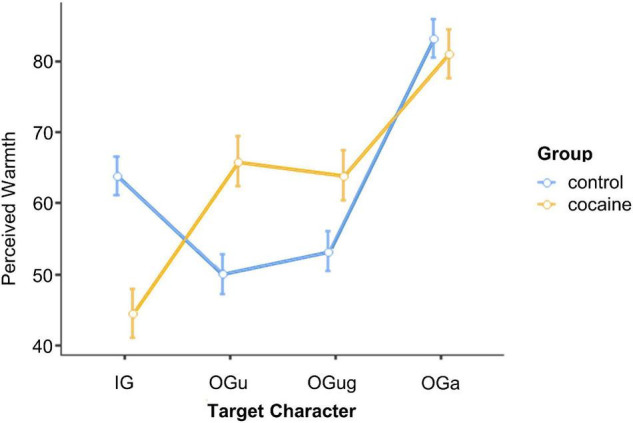
Perceived warmth of the different social targets. IG, in-group; OG_u_, out-group use; OG_ug_, out-group use + gender; OG_a_, out-group age. Error bars, standard errors.

By contrast, the ANOVA revealed a significant main effect of *Target Character*, *F*(3,177) = 31.76, *p* < 0.001, $\eta_{p}^{2}=0.35$, that was qualified by the significant interaction *Group* × *Target Character*, *F*(3,177) = 13.17, *p* < 0.001, $\eta_{p}^{2}=0.18$ (Figure 3). Both groups attributed the greatest warmth to the elderly out-group (OG_a_) characters [*p*-values < 0.004, for all (except two) pairwise comparisons (Tukey HSD) including the elderly character; *p* = 0.063 for comparison of OG_a_ vs. OG_u_ ratings in cocaine users; *p* = 0.997 for comparison of OG_a_ ratings for controls vs. cocaine users]. However, while the cocaine group rated the OG_u_ and OG_ug_ as warmer than their in-group (*p*-values ≤ 0.004), the control group showed, consistent with H1a, the opposite pattern (*p* = 0.005 for the comparison of IG and OG_u_; *p* = 0.079 for the comparison of IG and OG_ug_). Moreover, cocaine users evaluated the IG target as significantly colder than did the control group (*p* < 0.001), and their warmth ratings for both OG_u_, and OG_ug_ were higher than were the ratings for OG_u_ in the control group (*p*-values < 0.045). Neither group differentiated between OG_u_ and OG_ug_ (*p*-values > 0.987). In sum, therefore, leaving the elderly out-group aside, cocaine-consuming social targets were perceived as colder than non-cocaine consuming targets by both groups of participants. Moreover, inconsistent with our H1e, the data do not support the idea of cocaine users being characterized by limited variance in warmth attributions to different social targets. Finally, there was a significant main effect of *Gender*, *F*(1,59) = 10.74, *p* = 0.002, $\eta_{p}^{2}=0.15$, because females (*M* = 67.0, LCI = 63.8, UCI = 70.2) rated the social targets as warmer than did males (*M* = 59.5, LCI = 56.3, UCI = 62.6).

### Perceived Competence of the Different Social Targets

**FIGURE 4:**
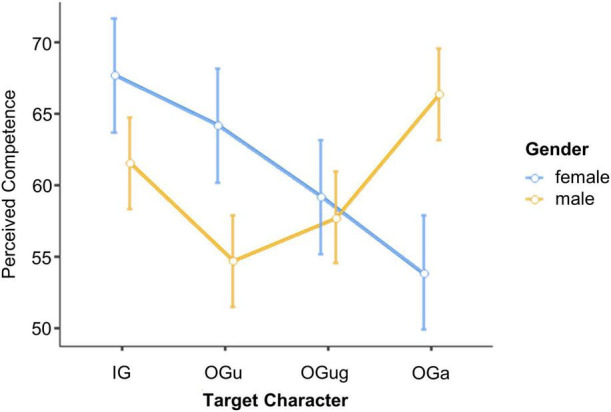
Perceived competence of the different social targets. IG, in-group; OG_u_, out-group use; OG_ug_, out-group use + gender; OG_a_, out-group age. Error bars, standard errors.

The repeated-measures ANOVA with the between-participants factors *Group* (cocaine and control) and *Gender* (male and female) and the within-participants factor *Target Character* (IG, OG_u_, OG_ug_, and OG_a_) yielded a significant interaction *Gender* × *Target Character*, *F*(3,177) = 4.01, *p* = 0.007, $\eta_{p}^{2}=0.06$ (cf. Figure 4). All *post hoc* pairwise comparisons (Tukey HSD) for this interaction failed to reach significance, and there was only a trend in male participants to attribute higher competence to the OG_a_ target than to the OG_u_ target (*p* = 0.090, *p*-values for the remaining pairwise comparisons >0.200). In sum, thus, the participants’ competence ratings are not in line with our hypotheses H1b, H1d, and H1e.

### Cognitive Empathy for the Different Social Targets

**FIGURE 5:**
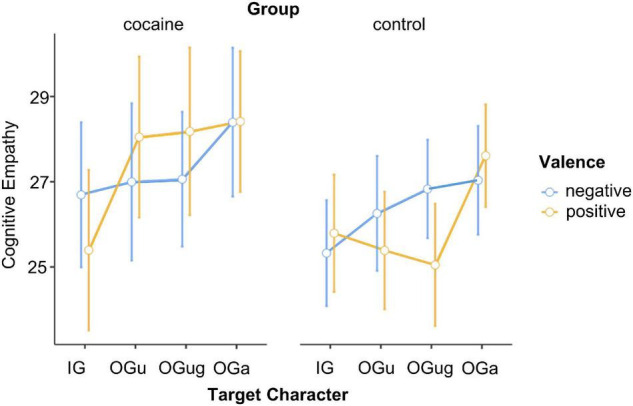
Cognitive empathy expressed for the different social targets. IG, in-group; OG_u_, out-group use; OG_ug_, out-group use + gender; OG_a_, out-group age. Error bars, standard errors.

A repeated-measures ANOVA with the between-participant factors *Group* (cocaine and control) and *Gender* (male and female) and the within-participants factors *Scenario Valence* (negative and positive) and *Target Character* (IG, OG_u_, OG_ug_, and OG_a_) was calculated on the participants’ cognitive empathy ratings. In line with our H2b, cocaine users and control participants did not differ in overall level of cognitive empathy, indexed by the non-significant main effect of *Group*, *F*(1,58) = 0.43, *p* = 0.517, $\eta_{p}^{2}=0.01$. The ANOVA showed a main effect of *Target Character*, *F*(3,174) = 6.11, *p* < 0.001, $\eta_{p}^{2}=0.10$, that was qualified by the interaction *Group* × *Scenario Valence* × *Target Character*, *F*(3,174) = 2.78, *p* = 0.043, $\eta_{p}^{2}=0.05$ (Figure 5).

To resolve this three-way interaction, we calculated separate ANOVAs (with the factors *Scenario Valence* and *Target Character*) for each level of *Group*. The ANOVA for control participants revealed a significant main effect of *Target Character*, *F*(3,111) = 3.23, *p* = 0.025, $\eta_{p}^{2}=0.08$, that was qualified by the interaction *Scenario Valence* × *Target Character*, *F*(3,111) = 3.59, *p* = 0.016, $\eta_{p}^{2}=0.09$. *Post hoc* Tukey tests revealed no significant differences between the target characters in the negative scenarios, which is consistent with H2a. By contrast, in the positive scenarios both same and other gender cocaine users (OG_u_ and OG_ug_) obtained (marginally) lower cognitive empathy ratings than did the OG_a_ (*p*-values = 0.067 and 0.012, respectively; *p*-values for the remaining *post hoc* Tukey tests related to this interaction >0.275). Overall, this result is consistent with our H2a. However, the fact that the in-group character differed neither from the cocaine-using targets nor from the elderly target is not in line with our assumption that the in-group character should evoke greater cognitive empathy than the cocaine-using targets. Hence, H2a is only partially supported by our data.

The ANOVA for cocaine users yielded a significant main effect of *Target Character*, *F*(3,60) = 6.14, *p* = 0.001, $\eta_{p}^{2}=0.24$. This effect arose because cocaine users attributed stronger feelings to the three out-group characters compared with the in-group character (*p*-values < 0.043), with no difference between the former (*p*-values > 0.446). There was further a main effect of *Gender*, *F*(1,20) = 7.93, *p* = 0.011, $\eta_{p}^{2}=0.28$, because female cocaine users displayed greater cognitive empathy (*M* = 30.7, LCI = 26.8, UCI = 34.6) than did male cocaine users (*M* = 22.9, LCI = 19.0, UCI = 26.8). Overall, cocaine users’ ratings (compared with control participants’ ratings) for cognitive empathy were not characterized by reduced differentiation between the social targets, thereby conflicting with our H2c. Yet, while control participants made a clear distinction between positive and negative scenarios, this was not the case in cocaine users.

### Emotional Empathy for the Different Social Targets

**FIGURE 6:**
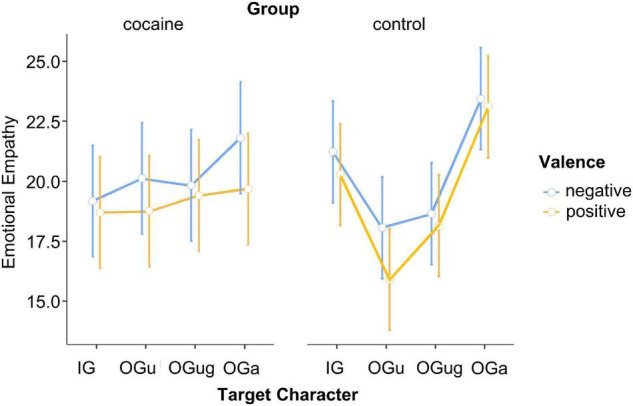
Emotional empathy expressed for the different social targets. IG, in-group; OG_u_, out-group use; OG_ug_, out-group use + gender; OG_a_, out-group age. Error bars, standard errors.

A repeated-measures ANOVA with the between-participants factors *Group* (cocaine and control) and *Gender* (male and female) and the within-participants factors *Scenario Valence* (negative and positive) and *Target Character* (IG, OG_u_, OG_ug_, and OG_a_) was calculated on the participants’ emotional empathy ratings. Inconsistent with H3b, cocaine users and control participants did not differ in overall level of emotional empathy displayed, shown by the non-significant main effect of *Group*, *F*(1,58) = 0.00, *p* = 0.950, $\eta_{p}^{2}=0.00$. The four-factorial ANOVA yielded a main effect of *Target Character*, *F*(3,174) = 5.07, *p* = 0.002, $\eta_{p}^{2}=0.08$, that was qualified by the interaction *Group* × *Target Character*, *F*(3,174) = 2.92, *p* = 0.036, $\eta_{p}^{2}=0.05$ (cf. Figure 6).

*Post hoc* Tukey tests for this interaction revealed greater emotional empathy attributed to the IG character than the OG_u_ character in the control participants (*p* = 0.037; consistent with H3a). Furthermore, the control participants rated their emotional empathy as higher for OG_a_ than for both OG_u_ and OG_ug_ (*p*-values ≤ 0.002; consistent with H3a). The remaining pairwise comparisons for this interaction did not reach significance (*p*-values > 0.407). Thus, while control participants more strongly emotionally empathized with IG and OG_a_ than with OG_u_ and OG_ug_, our cocaine users did not show any distinction between the social targets, which is supportive of H3c. Notably, this lack of differentiation was particularly strong in the cocaine users who were characterized by more enhanced consumption. Specifically, the lower the empathy score for IG – OG_u_, the higher were (a) frequency [cocaine use in times per week: Spearman’s rho (22) = −0.440, *p* = 0.040] and (b) doses [cocaine use in g per week: Spearman’s rho (22) = −0.469, *p* = 0.028] of cocaine consume. None of the effects involving *Scenario Valence* turned out significant. Hence, emotional empathy did not differ between negative and positive scenarios.

### Self-Related Future Expectancies

**FIGURE 7:**
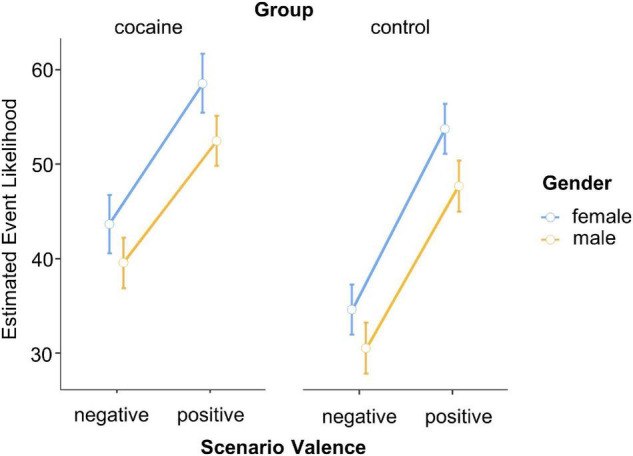
Self-related future expectancies. Error bars, standard errors.

The ANOVA with the between-participants factors *Group* (cocaine and control) and *Gender* (male and female) and the within-participants factor *Scenario Valence* (negative and positive) revealed a significant main effect of *Scenario Valence*, *F*(1,59) = 66.10, *p* < 0.001, $\eta_{p}^{2}=0.53$. As expected, participants displayed a strong optimism bias in that they attributed a greater likelihood to the occurrence of positive (*M* = 52.9%, LCI = 49.7%, UCI = 56.2%) rather than negative (*M* = 37.0%, LCI = 33.8%, UCI = 40.2%) scenarios (Figure 7). Notably, this effect was observed in both groups: absence of an interaction between *Group* and *Scenario Valence*, *F*(1,59) = 1.37, *p* = 0.247, $\eta_{p}^{2}=0.02$. Together, these observations are in line with H4a (existence of optimism bias in controls), but not with H4b (no altered optimism bias in cocaine users). Yet, *post hoc* Spearman correlations with consumption parameters in cocaine users revealed a positive correlation between the extent of optimism bias displayed and cocaine use in g per week, Spearman’s rho (22) = 0.543, *p* = 0.009. Additionally, we observed a significant main effect of *Group*, *F*(1,59) = 6.92, *p* = 0.011, $\eta_{p}^{2}=0.11$, relating to the fact that cocaine users (*M* = 48.6%, LCI = 44.8%, UCI = 52.3%) attributed overall greater likelihood to the occurrence of (both negative and positive) future events than did control participants (*M* = 41.4%, LCI = 37.6%, UCI = 45.1%).

## Discussion

The current study investigated whether person perception, cognitive and emotional empathy, as well as future expectancies are altered in cocaine users.

### Level of Identification (Manipulation Check)

As intended, our participants identified strongest with their respective in-group. Moreover, cocaine users and controls did not statistically differ in their overall level of identification with the target characters. Compared with controls, cocaine users also did not display reduced identification strength with the in-group, in particular. Consequently, the below findings cannot be explained by divergent levels of identification with the target characters in the current study.

### Perceived Warmth

Consistent with our H1a, controls attributed greater warmth to their in-group and the elderly targets than to the cocaine-using targets, displaying their stigmatization and discrimination of substance users (56). Such stigmatization may be problematic because it can feed back to the stigmatized group’s mental and physical health (57). By contrast, our H1c, specifying our expectation that cocaine users (compared with controls) would, overall, display lower ratings of warmth for the social targets did not reach support by the data at hand. The same was true for our H1e, which predicted little or no differentiation in warmth ratings between the social targets in cocaine users.

Cocaine users attributed lower warmth to the cocaine-using target (their in-group) than to the three non-cocaine-using targets. On the one hand, this result may speak to self-stigmatization of cocaine users [possibly resulting from their own public stigmatization; (56)]. Indeed, recent research [e.g., Crapanzano et al. (58)] has demonstrated that substance use disorder goes along with severe self-stigmatization processes, whose levels even surpass those revealed by individuals suffering from other mental illnesses [e.g., schizophrenia; (59)]. On the other hand, prior research has demonstrated reduced emotional empathy (8), lowered prosociality (10), and increased Utilitarian and Machiavellian tendencies (9) in cocaine use – wherefore our findings for warmth may alternatively or additionally map realistic person appraisals in both cocaine users and controls. If the latter were true, our results would hence speak to ego-syntonicity in cocaine users. In sum, therefore, both groups of participants rated the elderly target as warmest and the cocaine-consuming target(s) as colder than the non-cocaine-consuming targets. In addition, we observed higher warmth attributions in females than males, which may relate to females’ increased communal responsiveness (60).

### Perceived Competence

The first two hypotheses for competence (H1b: differing competence ratings for the four social targets in control participants, H1d: lower competence ratings in cocaine users than controls) were not supported by the existing data. By contrast, findings for our H1f were fully in line with our expectations in that cocaine users’ competence ratings did not distinguish between the different social targets. This could indeed speak to a tendency in cocaine users to reflect less about person characteristics in others. Yet, because control participants behaved in the same way, this result should not be over-interpreted. Unexpectedly, we observed a significant interaction *Target Character* × *Gender*, related to the trend in male participants to attribute particularly high competence to the elderly target. However, all *post hoc* tests failed to achieve significance, wherefore this effect clearly needs replication.

That we found only small effects in competence compared with warmth attributions is consistent with Abele and Wojciszke (61)’s theory regarding the impact of perspective taking on the ponderation of person characteristics. It may be assumed that participants in the current study adopted a so-called observer perspective and therefore weighted the targets’ competence less than their warmth (because another person’s warmth will serve the achievement of their own goals best). It will be interesting to study whether the opposite pattern will be observed when participants in an experiment adopt an actor perspective.

### Cognitive Empathy

Our cognitive empathy data suggest that potential discrimination and stigmatization of cocaine users by control participants in the current study may be limited to positive scenarios. Consistent with our H2a, for positive scenarios, controls expressed greatest cognitive empathy for the elderly target and lowest for cocaine-using targets. The in-group target was located in between. No such differentiation between the social targets was observed when negative scenarios were considered. This finding aligns with our earlier observation of reduced variation in cognitive empathy ratings for different social targets facing negative scenarios (17). The current data hence strengthen our earlier interpretation that people think that everybody has the right to feel bad and suffer. Such a point of view may have arisen from societal norms that rule how to empathize with others after those have experienced detrimental influences.

In line with our H2b, we found comparable overall levels of cognitive empathy in cocaine users and controls, suggesting that cocaine users are not characterized by generally impaired cognitive empathy. This observation harmonies with earlier findings revealing that cognitive empathy is not *per se* deviant in cocaine users (7, 8). Instead, there may exist specific impairments, expressed in reduced emotion recognition from prosody or integration of multiple emotional information sources (23).

Finally, the data at hand conflict with our H2c. Compared with the control participants’ cognitive empathy ratings, there was no indication of reduced variance in the cocaine users’ cognitive empathy ratings for the different social targets. Yet, the pattern of response in the two groups was different: cocaine users attributed weaker feelings to their in-group compared with all no substance using out-groups. This result possibly relates to blunted social reward processing in cocaine users (5). Whereas the general population tends to treat warm fellows (including their in-group) favorably across various domains (17, 42), this was not the case in our cocaine-using participants. Moreover, in the current study such unfavorable treatment of the in-group was observed across both types of scenarios, positive and negative. Contrary to the control group, the cocaine users therefore did not consider everybody to have the same right to feel bad.

### Emotional Empathy

In accordance with our H3a, control participants expressed the highest emotional empathy for the elderly and were least emotionally involved with the cocaine-using targets. Our results thus align with earlier findings in the area (17) and point once more to the stigmatization of substance users (56). Of note, our participants were not informed by personal characteristics or traits of any of the social targets under investigation. The sole mention of group membership was sufficient to provoke markedly different affective responses toward the social targets. Hence, our findings demonstrate the powerful influence of social classification, an influence that may turn out beneficial for members of some social groups (e.g., the elderly) but potentially damaging for members of other social groups [e.g., substance users such as cocaine users or alcoholics; (57)].

H3b, by contrast, was not supported by the data, because we did not find different overall levels of emotional empathy in cocaine users vs. controls. Hence, we did not replicate an earlier finding that revealed lowered emotional empathy in cocaine users (8). Yet, it has to be taken into consideration that the current task differed importantly from the Multifaceted Empathy Task [MET; (62)], which was employed in the earlier investigation. Whereas the MET involves emotionally-laden pictures that are presented to provoke emotional contagion, emotional perspective taking in the present task was more abstract, less automatic, and possibly characterized by higher cognitive and less emotional load.

Importantly, while the two groups of participants demonstrated a comparable level of overall emotional empathy, unlike the controls, cocaine users were characterized by a reduced differentiation between the social targets, which is consistent with H3c. A likely interpretation of this finding is that cocaine users are somewhat insensible or inattentive to social signals in their environment [including social stereotypes; see (5) for supportive evidence]. Of interest, our correlation analyses performed on the cocaine users’ empathy scores revealed that the favorability of the IG over OG_u_ varied as a negative function of the extent of their use of the drug. These data hence suggest that the degree of indifference reflected in emotional empathy directly relates to individual consumption patterns.

### Self-Related Expectancies

In line with our predictions (H4a), control participants displayed an optimism bias in that they imagined their future to be more positive than negative. Importantly, the included events had been judged for their likelihood of appearance in the general population in a previous study [see Dricu et al. (42), for details] and the average likelihood did not differ between positive and negative events. Therefore, our results cannot be explained by different base rates for positive vs. negative events in the general population.

We had further hypothesized that the size of the optimism bias would be altered in cocaine users (H4b). Yet, contrary to our expectations, the size of optimistic bias was comparable in cocaine users and controls. Notably, whereas there was no group difference revealed, our subsequently performed correlation analyses uncovered that optimism bias in cocaine users varied as a positive extent of dose levels. Thus, it is possible that participants with high doses more strongly benefited from enhancing effects of the drug. Interestingly, cocaine users rated the likelihood of both desirable and undesirable events higher than did controls, suggesting altered likelihood estimation *per se*. This observation may explain why cocaine users are bad decision makers also often taking higher risks [see (10, 32)] and possibly relates to lowered cortical thickness in the frontal cortex [(63), (64), see also (65), (66) for the frontal cortex’ involvement in cognitive estimation, prediction errors, and regulative actions].

## Limitations

We may be criticized because our study included a lower number of cocaine users than controls and an unequal distribution of males and females in the two groups of participants. We nonetheless believe that our results are valid because the prevalence of cocaine use is generally lower among females than males as revealed by the European Drug Report.^1^ Yet, because the overall sample size was comparably small for a cognitive study with chronic cocaine users, replication of the results is desired.

Furthermore, Table 2 revealed significant differences between cocaine users and controls on several clinical scales. ADHD-SR, BDI, and the SCID-II -borderline and antisocial personality disorder have been linked with cocaine use disorder before (4, 32, 67). It hence is possible that high scores on those scales are partially caused by using cocaine (or that these characteristics, in turn, influence the use of cocaine). Ideally, one would want to filter out the influences of such potentially confounding variables, i.e., by performing an analysis of covariance (ANCOVA). For two reasons, we decided not to conduct ANCOVAs with the relevant questionnaire scores as covariates. First, considering the low number of participants, the inclusion of any covariate would have reduced statistical power. Second, interpretation of ANVOCA results may be seriously compromised if the covariate and group membership are correlated [e.g., (68)]. The question of whether the personality characteristics influence drug consumption or vice versa cannot be addressed by the current study and requires future investigations. It thus remains to be determined whether the effects observed in the present research can be attributed to the consumption of the drug or to specific personality patterns prevailing in the cocaine users. Only a longitudinal study can address this question.

## Future Directions

The social phenomena investigated in cocaine use may be extended to the study of social optimism bias (42, 69–71). Because social expectancies are important for social interaction, it may pay off to identify critical social expectancies that should be corrected. Such an approach might permit cocaine users a more successful communication with others and support prevention of social isolation. In this context it will also be worthwhile to test for functional and structural particularities (43, 44, 72) that are likely associated with altered social expectancies in substance use disorders. Furthermore, substance-naïve individuals have been demonstrated to hold rather pessimistic future expectancies for substance users (42, 71, 72). Such overpessimistic expectancies may require modification to reduce stigmatization and discrimination of cocaine users by the general population (56).

Finally, our data compared with earlier findings suggest that the revelation of emotional empathy impairments in cocaine use is somewhat dependent on the task. In future examinations, the same sample of participants (users and controls) should therefore undergo different experimental paradigms (e.g., MET and the current paradigm) to permit better identification of the specific facets of emotional empathy that are impaired.

## Summary and Conclusion

We did not find differences in social identification (manipulation check) between cocaine users and controls, suggesting that such automatic and basic social processing is not flawed in cocaine users. However, we observed that cocaine users (compared with substance-naïve individuals) attribute lower warmth to people they feel alike. Moreover, they see non-consuming individuals as warmer and more likeable than they see people, who are like themselves. That the in-group is suchlike debased is rather uncommon and may point to massively compromised self-value and self-esteem resulting from (self-)stigmatization. Comparably, we observed no in-group preference in the cocaine users’ emotional empathy ratings. An in-group that is evaluated as more unlikeable than diverse out-groups may not trigger enhanced affective sharing, which is typically elicited once we see similar others in emotional situations. Our data further suggest that such deviance might be a direct consequence of a user’s consumption pattern – or vice versa. Together, our findings point to multiple interdependencies between (a) personal factors (e.g., cocaine users’ perception of the self and others), (b) the external environment (social distancing from and stigmatization of cocaine users by substance-naïve individuals), and (c) substance-related behavior (cocaine intake), which is fully in line with recently suggested reciprocal determinism and metacontingencies in addiction (73). Future interventions should hence address critical (self-)stigmatization processes to break the vicious circle of mutual social distancing and increased dedication to the drug. Finally, self-related future expectancies are not *per se* more negative or positive in cocaine users compared with controls. Yet, it remains to be determined whether there are peculiarities when it comes to social future expectancies.

## Data Availability Statement

The raw data supporting the conclusions of this article will be made available by the authors, without undue reservation.

## Ethics Statement

The studies involving human participants were reviewed and approved by the Ethics Committee of the Canton Zürich. The patients/participants provided their written informed consent to participate in this study.

## Author Contributions

TA, LS, and BQ developed the study concept and design. A-KK and BK-S conducted the assessments. SB and BK-S programmed the experiment. A-KK curated the data and wrote sections of the manuscript. TA performed the statistical analysis and wrote the first draft of the manuscript. MB conducted hair analyses and supported data interpretation. LS supported data interpretation. All authors contributed to manuscript revision, read, and approved the submitted version.

## Conflict of Interest

The authors declare that the research was conducted in the absence of any commercial or financial relationships that could be construed as a potential conflict of interest.

## Publisher’s Note

All claims expressed in this article are solely those of the authors and do not necessarily represent those of their affiliated organizations, or those of the publisher, the editors and the reviewers. Any product that may be evaluated in this article, or claim that may be made by its manufacturer, is not guaranteed or endorsed by the publisher.

## Footnotes

1
https://www.emcdda.europa.eu/publications/edr/trends-developments/2021_en
