# Supplementary material for: In- and Out-Group Effects on Social Perception and Empathy in Cocaine Use Disorder
Source: Front Psychiatry. 2022 Aug 1;13:879016. doi: 10.3389/fpsyt.2022.879016 (PMC9376468; doi:10.3389/fpsyt.2022.879016)
Supplement: Supplementary file 1 [file Table_1.DOCX]

**Supplementary Material**

1. **Material and Methods**

**1A. Level of Identification with the different social targets (manipulation check; Suppl. Fig. 1)**


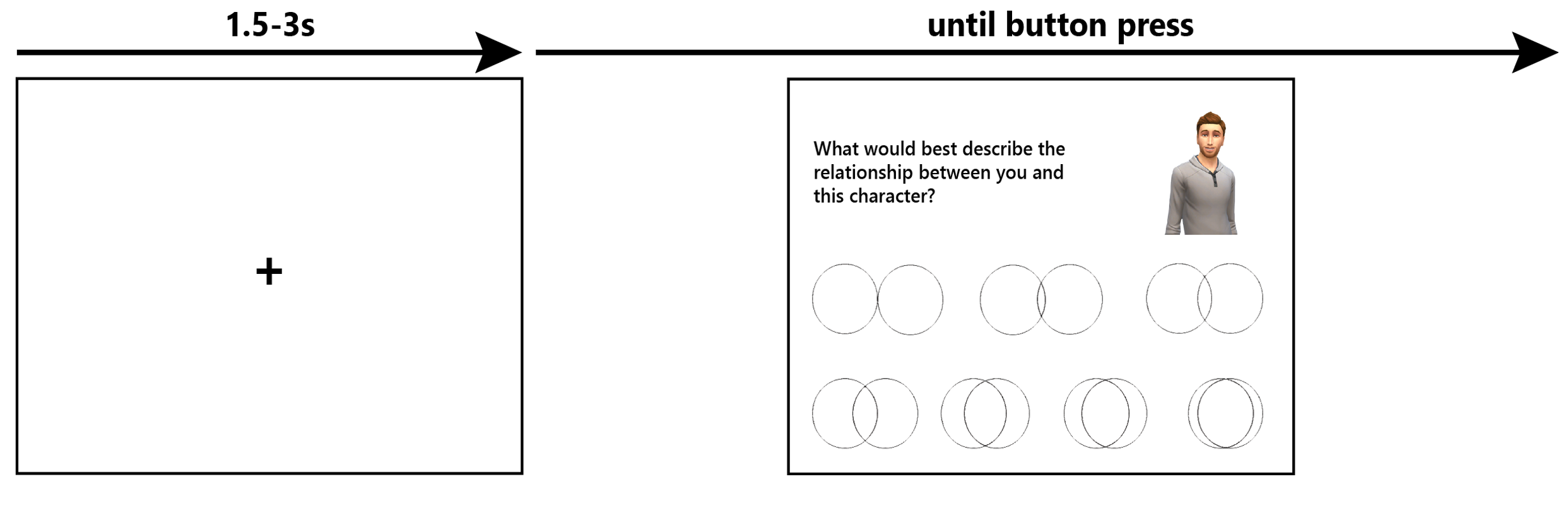

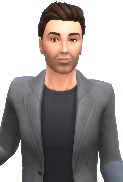

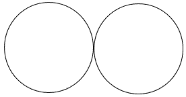

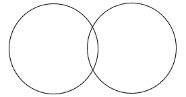

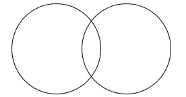

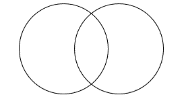

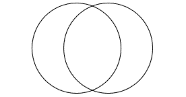

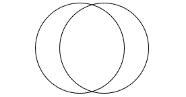

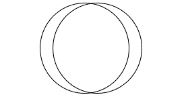


**1B. Assessment of perceived warmth and competence (Suppl. Fig. 2)**


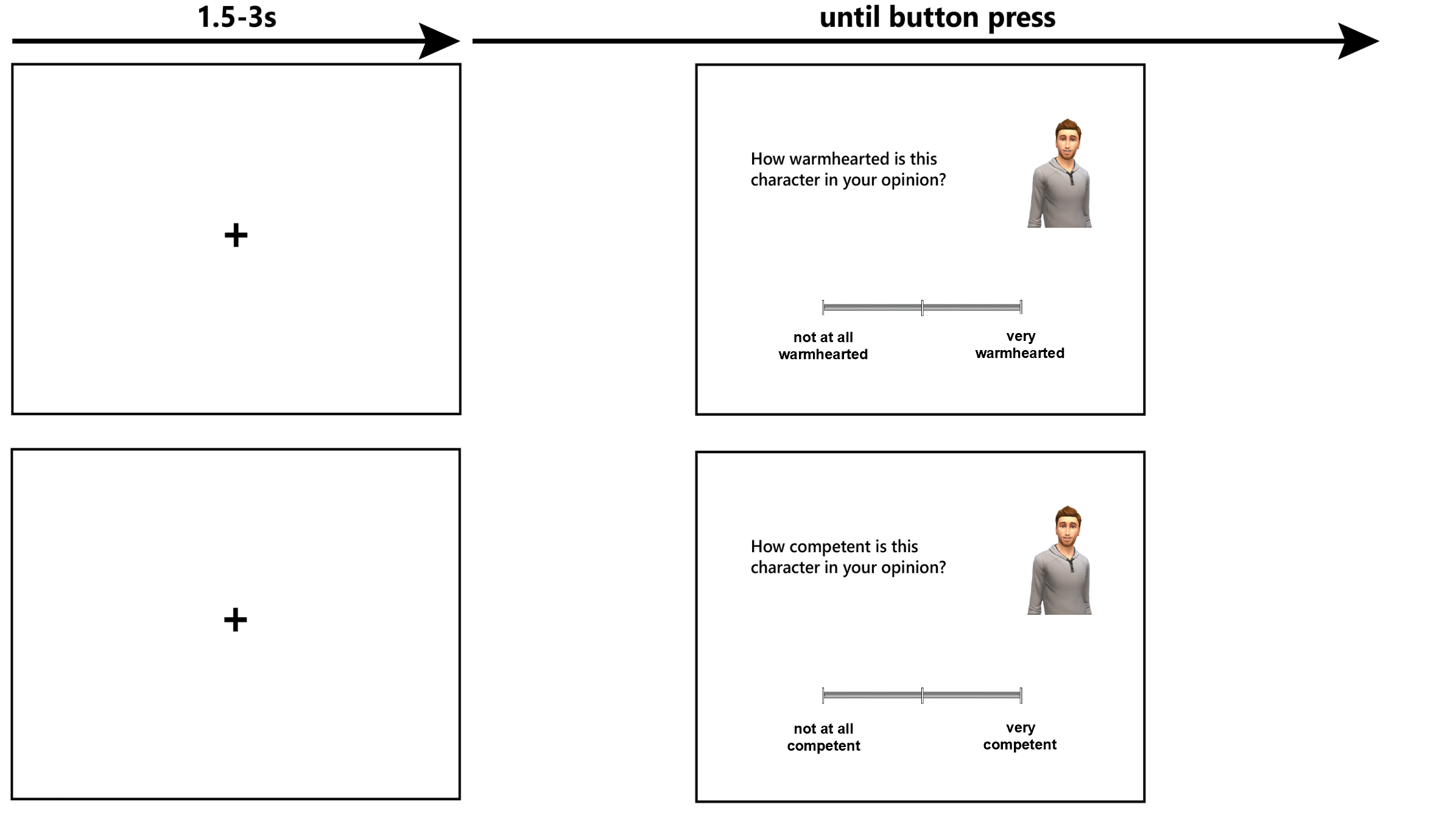

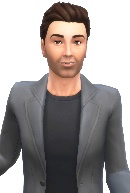

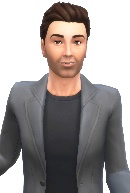

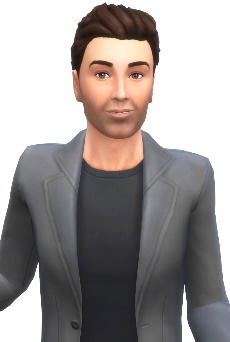

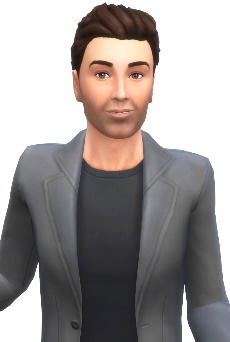


**1C. Assessment of cognitive and emotional empathy (Suppl. Fig. 3)**


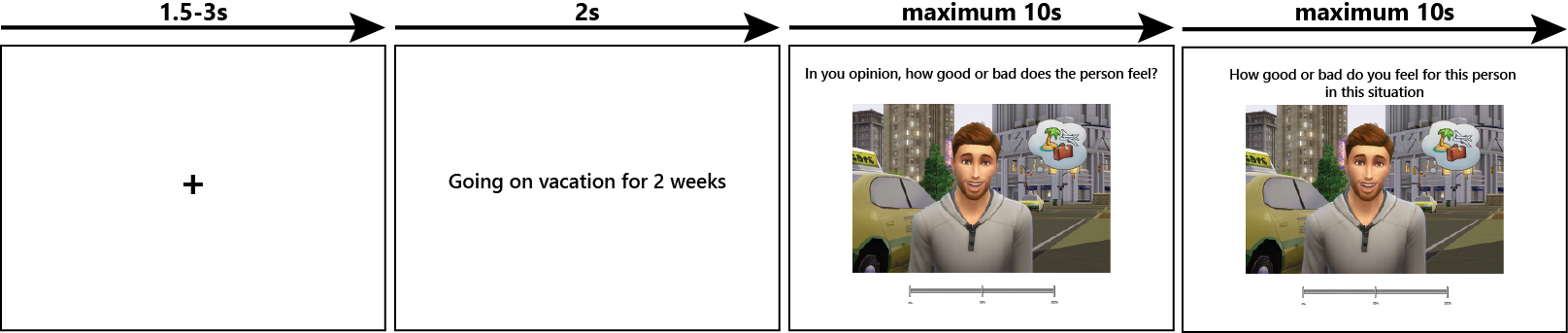


**very bad**

**very good**

**very bad**

**very good**

Cognitive empathy

Emotional empathy

**very bad**

**very good**

**very bad**

**very good**

**Bumping into an old friend on the street**

In your opinion, how good or

bad does the person feel?

How good or bad do you feel

for this person?


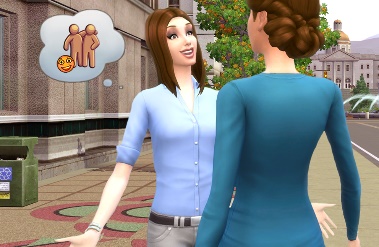

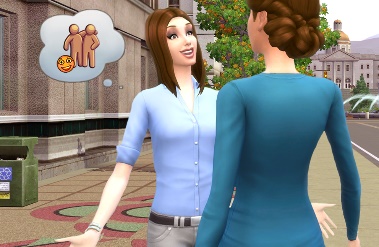


**1D. List of scenarios used in the empathy and self-related expectancy tasks**

| Scenario | Valence |
| --- | --- |
| **The store closes just as you arrive** | Negative |
| **Drinking cold coffee/tea** | Negative |
| **Being left by the partner** | Negative |
| **Computer crashes in the middle of writing a text** | Negative |
| **Lose 50 CHF** | Negative |
| **Receiving a wound that needs to be sewn** | Negative |
| **Being falsely accused of a serious crime** | Negative |
| **Getting bitten by a dog** | Negative |
| **Being hugged** | Positive |
| **Enjoy a warm bath or shower on a cold day** | Positive |
| **New neighbor comes over to introduce themselves** | Positive |
| **Bumping into an old friend on the street** | Positive |
| **Delivering a speech successfully** | Positive |
| **Writing a bestseller about one’s own life** | Positive |
| **Find a 50 CHF bill on the ground** | Positive |
| **Seeing a comet in the sky** | Positive |

1. **Pattern of correlations between the different social constructs**

**2A. Cocaine users: Across all scenarios**

|  |  |  |  |  |  |  |  |  |  |  |
| --- | --- | --- | --- | --- | --- | --- | --- | --- | --- | --- |
| **In-group** |  |  | Cognitive Empathy |  | Emotional Empathy |  | Social Identification |  | Perceived Warmth | |
|  | Emotional Empathy | r | **.592** | ** |  |  |  |  |  |  |
|  |  | p | **.004** |  |  |  |  |  |  |  |
|  | Social Identification | r | .208 |  | .088 |  |  |  |  |  |
|  |  | p | .352 |  | .679 |  |  |  |  |  |
|  | Perceived Warmth | r | .008 |  | .008 |  | -.054 |  |  |  |
|  |  | p | .971 |  | .973 |  | .811 |  |  |  |
|  | Perceived Competence | r | .096 |  | .019 |  | -.196 |  | -.046 |  |
|  |  | p | .670 |  | .933 |  | .381 |  | .837 |  |
| **Out-group use** |  |  | Cognitive Empathy |  | Emotional Empathy |  | Social Identification |  | Perceived Warmth | |
|  | Emotional Empathy | r | **.669** | *** |  |  |  |  |  |  |
|  |  | p | **< .001** |  |  |  |  |  |  |  |
|  | Social Identification | r | -.011 |  | .241 |  |  |  |  |  |
|  |  | p | .962 |  | .280 |  |  |  |  |  |
|  | Perceived Warmth | r | .405 |  | .086 |  | .067 |  |  |  |
|  |  | p | .062 |  | .703 |  | .768 |  |  |  |
|  | Perceived Competence | r | .325 |  | .298 |  | .045 |  | **.555** | ** |
|  |  | p | .140 |  | .178 |  | .842 |  | **.007** |  |
| **Out-group use+gender** |  |  | Cognitive Empathy |  | Emotional Empathy |  | Social Identification |  | Perceived Warmth | |
|  | Emotional Empathy | r | **.544** | ** |  |  |  |  |  |  |
|  |  | p | .**009** |  |  |  |  |  |  |  |
|  | Social Identification | r | -.044 |  | .148 |  |  |  |  |  |
|  |  | p | .846 |  | .511 |  |  |  |  |  |
|  | Perceived Warmth | r | **.452** | * | .169 |  | -.042 |  |  |  |
|  |  | p | .**035** |  | .453 |  | .851 |  |  |  |
|  | Perceived Competence | r | .302 |  | .299 |  | .084 |  | .346 |  |
|  |  | p | .172 |  | .177 |  | .710 |  | .114 |  |
| **Out-group age** |  |  | Cognitive Empathy |  | Emotional Empathy |  | Social Identification |  | Perceived Warmth | |
|  | Emotional Empathy | r | **.553** | ** |  |  |  |  |  |  |
|  |  | p | **.008** |  |  |  |  |  |  |  |
|  | Social Identification | r | .078 |  | .302 |  |  |  |  |  |
|  |  | p | .730 |  | .172 |  |  |  |  |  |
|  | Perceived Warmth | r | **.557** | ** | **.540** | ** | .067 |  |  |  |
|  |  | p | **.007** |  | **.001** |  | .768 |  |  |  |
|  | Perceived Competence | r | -.127 |  | .195 |  | .117 |  | .114 |  |
|  |  | p | .573 |  | .384 |  | .604 |  | .613 |  |

*Note*. **p* < .05, ***p* < .01, ****p* < .001. *n* = 22 participants.

**2B.** **Cocaine users: For negative scenarios**

|  |  |  |  |  |  |  |  |  |  |  |
| --- | --- | --- | --- | --- | --- | --- | --- | --- | --- | --- |
| **In-group** |  |  | Cognitive Empathy |  | Emotional Empathy |  | Social Identification |  | Perceived Warmth | |
|  | Emotional Empathy | r | **.537** | * |  |  |  |  |  |  |
|  |  | p | **.010** |  |  |  |  |  |  |  |
|  | Social Identification | r | .355 |  | .136 |  |  |  |  |  |
|  |  | p | .105 |  | .547 |  |  |  |  |  |
|  | Perceived Warmth | r | .197 |  | .093 |  | -.054 |  |  |  |
|  |  | p | .379 |  | .682 |  | .811 |  |  |  |
|  | Perceived Competence | r | -.125 |  | -.023 |  | -.196 |  | -.046 |  |
|  |  | p | .580 |  | .917 |  | .381 |  | .837 |  |
| **Out-group use** |  |  | Cognitive Empathy |  | Emotional Empathy |  | Social Identification |  | Perceived Warmth | |
|  | Emotional Empathy | r | **.524** | * |  |  |  |  |  |  |
|  |  | p | .**012** |  |  |  |  |  |  |  |
|  | Social Identification | r | -.010 |  | .136 |  |  |  |  |  |
|  |  | p | .964 |  | .547 |  |  |  |  |  |
|  | Perceived Warmth | r | .373 |  | -.075 |  | .067 |  |  |  |
|  |  | p | .087 |  | .741 |  | .768 |  |  |  |
|  | Perceived Competence | r | .219 |  | .179 |  | .045 |  | **.555** | ** |
|  |  | p | .327 |  | .426 |  | .842 |  | **.007** |  |
| **Out-group use+gender** |  |  | Cognitive Empathy |  | Emotional Empathy |  | Social Identification |  | Perceived Warmth | |
|  | Emotional Empathy | r | .320 |  |  |  |  |  |  |  |
|  |  | p | .146 |  |  |  |  |  |  |  |
|  | Social Identification | r | .028 |  | .133 |  |  |  |  |  |
|  |  | p | .902 |  | .555 |  |  |  |  |  |
|  | Perceived Warmth | r | .338 |  | .139 |  | -.042 |  |  |  |
|  |  | p | .124 |  | .537 |  | .851 |  |  |  |
|  | Perceived Competence | r | .305 |  | .145 |  | .084 |  | .346 |  |
|  |  | p | .168 |  | .519 |  | .710 |  | .114 |  |
| **Out-group age** |  |  | Cognitive Empathy |  | Emotional Empathy |  | Social Identification |  | Perceived Warmth | |
|  | Emotional Empathy | r | .358 |  |  |  |  |  |  |  |
|  |  | p | .102 |  |  |  |  |  |  |  |
|  | Social Identification | r | .050 |  | .266 |  |  |  |  |  |
|  |  | p | .824 |  | .231 |  |  |  |  |  |
|  | Perceived Warmth | r | .382 |  | **.512** | * | .067 |  |  |  |
|  |  | p | .079 |  | **.015** |  | .768 |  |  |  |
|  | Perceived Competence | r | -.168 |  | .126 |  | .117 |  | .114 |  |
|  |  | p | .454 |  | .577 |  | .604 |  | .613 |  |

*Note*. **p* < .05, ***p* < .01. *n* = 22 participants.

**2C. Cocaine users: For positive scenarios**

|  |  |  |  |  |  |  |  |  |  |  |
| --- | --- | --- | --- | --- | --- | --- | --- | --- | --- | --- |
| **In-group** |  |  | Cognitive Empathy |  | Emotional Empathy |  | Social Identification |  | Perceived Warmth | |
|  | Emotional Empathy | r | **.690** | *** |  |  |  |  |  |  |
|  |  | p | **< .001** |  |  |  |  |  |  |  |
|  | Social Identification | r | .034 |  | .012 |  |  |  |  |  |
|  |  | p | .880 |  | .957 |  |  |  |  |  |
|  | Perceived Warmth | r | -.117 |  | -.087 |  | -.054 |  |  |  |
|  |  | p | .604 |  | .701 |  | .811 |  |  |  |
|  | Perceived Competence | r | .203 |  | .060 |  | -.196 |  | -.046 |  |
|  |  | p | .366 |  | .791 |  | .381 |  | .837 |  |
| **Out-group use** |  |  | Cognitive Empathy |  | Emotional Empathy |  | Social Identification |  | Perceived Warmth | |
|  | Emotional Empathy | r | **.711** | *** |  |  |  |  |  |  |
|  |  | p | **< .001** |  |  |  |  |  |  |  |
|  | Social Identification | r | -.009 |  | .302 |  |  |  |  |  |
|  |  | p | .967 |  | .173 |  |  |  |  |  |
|  | Perceived Warmth | r | .368 |  | .221 |  | .067 |  |  |  |
|  |  | p | .092 |  | .324 |  | .768 |  |  |  |
|  | Perceived Competence | r | .370 |  | .362 |  | .045 |  | **.555** | ** |
|  |  | p | .090 |  | .097 |  | .842 |  | **.007** |  |
| **Out-group use+gender** |  |  | Cognitive Empathy |  | Emotional Empathy |  | Social Identification |  | Perceived Warmth | |
|  | Emotional Empathy | r | **.596** | ** |  |  |  |  |  |  |
|  |  | p | **.003** |  |  |  |  |  |  |  |
|  | Social Identification | r | -.081 |  | .146 |  |  |  |  |  |
|  |  | p | .719 |  | .517 |  |  |  |  |  |
|  | Perceived Warmth | r | **.426** | * | .177 |  | -.042 |  |  |  |
|  |  | p | **.048** |  | .430 |  | .851 |  |  |  |
|  | Perceived Competence | r | **.232** |  | .407 |  | .084 |  | .346 |  |
|  |  | p | .299 |  | .060 |  | .710 |  | .114 |  |
| **Out-group age** |  |  | Cognitive Empathy |  | Emotional Empathy |  | Social Identification |  | Perceived Warmth | |
|  | Emotional Empathy | r | **.636** | ** |  |  |  |  |  |  |
|  |  | p | **.001** |  |  |  |  |  |  |  |
|  | Social Identification | r | .092 |  | .308 |  |  |  |  |  |
|  |  | p | .684 |  | .163 |  |  |  |  |  |
|  | Perceived Warmth | r | **.636** | ** | **.513** | * | .067 |  |  |  |
|  |  | p | **.001** |  | **.015** |  | .768 |  |  |  |
|  | Perceived Competence | r | -.069 |  | .246 |  | .117 |  | .114 |  |
|  |  | p | .759 |  | .269 |  | .604 |  | .613 |  |

*Note*. **p* < .05, ***p* < .01, ****p* < .001. *n* = 22 participants.

**2D. Controls: Across all scenarios**

|  |  |  |  |  |  |  |  |  |  |  |
| --- | --- | --- | --- | --- | --- | --- | --- | --- | --- | --- |
| **In-group** |  |  | Cognitive Empathy |  | Emotional Empathy |  | Social Identification |  | Perceived Warmth | |
|  | Emotional Empathy | r | **.658** | *** |  |  |  |  |  |  |
|  |  | p | **< .001** |  |  |  |  |  |  |  |
|  | Social Identification | r | .034 |  | .076 |  |  |  |  |  |
|  |  | p | .838 |  | .646 |  |  |  |  |  |
|  | Perceived Warmth | r | .101 |  | .125 |  | **.372** | * |  |  |
|  |  | p | .540 |  | .448 |  | **.018** |  |  |  |
|  | Perceived Competence | r | .061 |  | .251 |  | .245 |  | **.493** |  |
|  |  | p | .712 |  | .123 |  | .127 |  | **.001** |  |
| **Out-group use** |  |  | Cognitive Empathy |  | Emotional Empathy |  | Social Identification |  | Perceived Warmth | |
|  | Emotional Empathy | r | **.409** | ** |  |  |  |  |  |  |
|  |  | p | **.010** |  |  |  |  |  |  |  |
|  | Social Identification | r | .144 |  | .147 |  |  |  |  |  |
|  |  | p | .383 |  | .372 |  |  |  |  |  |
|  | Perceived Warmth | r | .140 |  | .135 |  | **.400** | * |  |  |
|  |  | p | .395 |  | .413 |  | **.010** |  |  |  |
|  | Perceived Competence | r | -.055 |  | .058 |  | .204 |  | **.423** | ** |
|  |  | p | .742 |  | .726 |  | .207 |  | **.007** |  |
| **Out-group use+gender** |  |  | Cognitive Empathy |  | Emotional Empathy |  | Social Identification |  | Perceived Warmth | |
|  | Emotional Empathy | r | **.587** | *** |  |  |  |  |  |  |
|  |  | p | **< .001** |  |  |  |  |  |  |  |
|  | Social Identification | r | .077 |  | .166 |  |  |  |  |  |
|  |  | p | .641 |  | .312 |  |  |  |  |  |
|  | Perceived Warmth | r | -.225 |  | -.311 |  | .098 |  |  |  |
|  |  | p | .168 |  | .054 |  | .546 |  |  |  |
|  | Perceived Competence | r | -.024 |  | -.173 |  | .030 |  | **.453** | ** |
|  |  | p | .885 |  | .293 |  | .857 |  | **.003** |  |
| **Out-group age** |  |  | Cognitive Empathy |  | Emotional Empathy |  | Social Identification |  | Perceived Warmth | |
|  | Emotional Empathy | r | **.653** | *** |  |  |  |  |  |  |
|  |  | p | **< .001** |  |  |  |  |  |  |  |
|  | Social Identification | r | .192 |  | .197 |  |  |  |  |  |
|  |  | p | .243 |  | .229 |  |  |  |  |  |
|  | Perceived Warmth | r | .249 |  | .149 |  | .247 |  |  |  |
|  |  | p | .126 |  | .364 |  | .124 |  |  |  |
|  | Perceived Competence | r | .091 |  | .183 |  | .310 |  | -.008 |  |
|  |  | p | .582 |  | .264 |  | .051 |  | .961 |  |

*Note*. **p* < .05, ***p* < .01, ****p* < .001. *n* = 40 participants; for correlations involving the empathy measures: n = 39 participants.

**2E. Controls: For negative scenarios**

|  |  |  |  |  |  |  |  |  |  |  |
| --- | --- | --- | --- | --- | --- | --- | --- | --- | --- | --- |
| **In-group** |  |  | Cognitive Empathy |  | Emotional Empathy |  | Social Identification |  | Perceived Warmth | |
|  | Emotional Empathy | r | **.591** | *** |  |  |  |  |  |  |
|  |  | p | **< .001** |  |  |  |  |  |  |  |
|  | Social Identification | r | .100 |  | .136 |  |  |  |  |  |
|  |  | p | .546 |  | .409 |  |  |  |  |  |
|  | Perceived Warmth | r | .150 |  | .233 |  | **.372** | * |  |  |
|  |  | p | .362 |  | .153 |  | **.018** |  |  |  |
|  | Perceived Competence | r | .145 |  | **.350** | ****** | .245 |  | **.493** | ** |
|  |  | p | .380 |  | **.029** |  | .127 |  | **.001** |  |
| **Out-group use** |  |  | Cognitive Empathy |  | Emotional Empathy |  | Social Identification |  | Perceived Warmth | |
|  | Emotional Empathy | r | **.385** | ***** |  |  |  |  |  |  |
|  |  | p | **.015** |  |  |  |  |  |  |  |
|  | Social Identification | r | .064 |  | .166 |  |  |  |  |  |
|  |  | p | .698 |  | .311 |  |  |  |  |  |
|  | Perceived Warmth | r | .016 |  | .115 |  | **.400** | * |  |  |
|  |  | p | .923 |  | .487 |  | **.010** |  |  |  |
|  | Perceived Competence | r | -.130 |  | .057 |  | .204 |  | **.423** | ** |
|  |  | p | .430 |  | .732 |  | .207 |  | **.007** |  |
| **Out-group use+gender** |  |  | Cognitive Empathy |  | Emotional Empathy |  | Social Identification |  | Perceived Warmth | |
|  | Emotional Empathy | r | **.525** | *** |  |  |  |  |  |  |
|  |  | p | **< .001** |  |  |  |  |  |  |  |
|  | Social Identification | r | .045 |  | .076 |  |  |  |  |  |
|  |  | p | .786 |  | .647 |  |  |  |  |  |
|  | Perceived Warmth | r | -.258 |  | -.288 |  | .098 |  |  |  |
|  |  | p | .112 |  | .075 |  | .546 |  |  |  |
|  | Perceived Competence | r | -.116 |  | -.111 |  | .030 |  | **.453** | ** |
|  |  | p | .481 |  | .502 |  | .857 |  | **.003** |  |
| **Out-group age** |  |  | Cognitive Empathy |  | Emotional Empathy |  | Social Identification |  | Perceived Warmth | |
|  | Emotional Empathy | r | .632 | *** |  |  |  |  |  |  |
|  |  | p | < .001 |  |  |  |  |  |  |  |
|  | Social Identification | r | .238 |  | .172 |  |  |  |  |  |
|  |  | p | .144 |  | .296 |  |  |  |  |  |
|  | Perceived Warmth | r | .300 |  | .148 |  | .247 |  |  |  |
|  |  | p | .063 |  | .368 |  | .124 |  |  |  |
|  | Perceived Competence | r | .052 |  | .164 |  | .310 |  | -.008 |  |
|  |  | p | .752 |  | .317 |  | .051 |  | .961 |  |

*Note*. **p* < .05, ***p* < .01, ****p* < .001. *n* = 40 participants; for correlations involving the empathy measures: n = 39 participants.

**2F. Controls: For positive scenarios**

|  |  |  |  |  |  |  |  |  |  |  |
| --- | --- | --- | --- | --- | --- | --- | --- | --- | --- | --- |
| **In-group** |  |  | Cognitive Empathy |  | Emotional Empathy |  | Social Identification |  | Perceived Warmth | |
|  | Emotional Empathy | r | **.599** | *** |  |  |  |  |  |  |
|  |  | p | **< .001** |  |  |  |  |  |  |  |
|  | Social Identification | r | -.041 |  | .009 |  |  |  |  |  |
|  |  | p | .802 |  | .955 |  |  |  |  |  |
|  | Perceived Warmth | r | .037 |  | .007 |  | **.372** | * |  |  |
|  |  | p | .824 |  | .967 |  | **.018** |  |  |  |
|  | Perceived Competence | r | -.037 |  | .127 |  | .245 |  | **.493** | ** |
|  |  | p | .825 |  | .443 |  | .127 |  | **.001** |  |
| **Out-group use** |  |  | Cognitive Empathy |  | Emotional Empathy |  | Social Identification |  | Perceived Warmth | |
|  | Emotional Empathy | r | **.390** | * |  |  |  |  |  |  |
|  |  | p | **.014** |  |  |  |  |  |  |  |
|  | Social Identification | r | .201 |  | .114 |  |  |  |  |  |
|  |  | p | .219 |  | .489 |  |  |  |  |  |
|  | Perceived Warmth | r | .242 |  | .143 |  | **.400** | * |  |  |
|  |  | p | .137 |  | .384 |  | **.010** |  |  |  |
|  | Perceived Competence | r | .027 |  | .054 |  | .204 |  | **.423** | ** |
|  |  | p | .868 |  | .743 |  | .207 |  | **.007** |  |
| **Out-group use+gender** |  |  | Cognitive Empathy |  | Emotional Empathy |  | Social Identification |  | Perceived Warmth | |
|  | Emotional Empathy | r | **.521** | *** |  |  |  |  |  |  |
|  |  | p | **< .001** |  |  |  |  |  |  |  |
|  | Social Identification | r | .100 |  | .218 |  |  |  |  |  |
|  |  | p | .545 |  | .182 |  |  |  |  |  |
|  | Perceived Warmth | r | -.179 |  | -.265 |  | .098 |  |  |  |
|  |  | p | .276 |  | .103 |  | .546 |  |  |  |
|  | Perceived Competence | r | .059 |  | -.195 |  | .030 |  | **.453** | ** |
|  |  | p | .719 |  | .233 |  | .857 |  | **.003** |  |
| **Out-group age** |  |  | Cognitive Empathy |  | Emotional Empathy |  | Social Identification |  | Perceived Warmth | |
|  | Emotional Empathy | r | **.658** | *** |  |  |  |  |  |  |
|  |  | p | **< .001** |  |  |  |  |  |  |  |
|  | Social Identification | r | .121 |  | .207 |  |  |  |  |  |
|  |  | p | .463 |  | .206 |  |  |  |  |  |
|  | Perceived Warmth | r | .169 |  | .138 |  | .247 |  |  |  |
|  |  | p | .305 |  | .402 |  | .124 |  |  |  |
|  | Perceived Competence | r | .125 |  | .188 |  | .310 |  | -.008 |  |
|  |  | p | .448 |  | .252 |  | .051 |  | .961 |  |

*Note*. **p* < .05, ***p* < .01, ****p* < .001. *n* = 40 participants; for correlations involving the empathy measures: n = 39 participants.

1. **ANOVA results for the different dependent variables**

**3A. Similarity ratings (IOS Scale, manipulation check)**

| Within Subjects Effects | | | | | | | | | | | | | | | | | | | | | | | | | | | |
| --- | --- | --- | --- | --- | --- | --- | --- | --- | --- | --- | --- | --- | --- | --- | --- | --- | --- | --- | --- | --- | --- | --- | --- | --- | --- | --- | --- |
|  | | | |  |  | | | |  |  | | |  |  | | | |  | |  | |  | |  |  |  |  |
|  | | | | | **Sum of Squares** | | | | | **df** | | | | **Mean Square** | | | | | | **F** | | | | **p** | | **η²_p_** | |
| Target Character | | | |  | 114.10 | | | |  | 3 | | |  | 38.03 | | | |  | | 17.74 | |  | | < .001 |  | 0.231 |  |
| Target Character ✻ Group | | | |  | 10.93 | | | |  | 3 | | |  | 3.64 | | | |  | | 1.70 | |  | | 0.169 |  | 0.028 |  |
| Target Character ✻ Gender | | | |  | 8.80 | | | |  | 3 | | |  | 2.93 | | | |  | | 1.37 | |  | | 0.254 |  | 0.023 |  |
| Residual | | | |  | 379.46 | | | |  | 177 | | |  | 2.14 | | | |  | |  | |  | |  |  |  |  |
| Note. Type 3 Sums of Squares | | | | | | | | | | | | | | | | | | | | | | | | | | | |
|  | | | | | | | | | | | | | | | | | | | | | | | | | | | |
| Between Subjects Effects | | | | | | | | | | | | | | | | | | | | | | |  |  |  |  |  |
|  |  |  |  | | |  |  |  | | |  |  | | |  |  |  | |  | |  | |  |  |  |  |  |
|  | | **Sum of Squares** | | | | **df** | | **Mean Square** | | | | **F** | | | | **p** | | | **η²_p_** | | | |  |  |  |  |  |
| Group |  | 9.58 |  | | | 1 |  | 9.58 | | |  | 3.06 | | |  | 0.085 |  | | 0.049 | |  | |  |  |  |  |  |
| Gender |  | 20.21 |  | | | 1 |  | 20.21 | | |  | 6.47 | | |  | 0.014 |  | | 0.099 | |  | |  |  |  |  |  |
| Residual |  | 184.44 |  | | | 59 |  | 3.13 | | |  |  | | |  |  |  | |  | |  | |  |  |  |  |  |
| Note. Type 3 Sums of Squares | | | | | | | | | | | | | | | | | | | | | | |  |  |  |  |  |
|  | | | | | | | | | | | | | | | | | | | | | | |  |  |  |  |  |

**3B. Perceived warmth**

| Within Subjects Effects | | | | | | | | | | | | | |
| --- | --- | --- | --- | --- | --- | --- | --- | --- | --- | --- | --- | --- | --- |
|  |  |  |  |  |  |  |  |  |  |  |  |  |  |
|  | | **Sum of Squares** | | **df** | | **Mean Square** | | **F** | | **p** | | **η²_p_** | |
| Target Character |  | 24851 |  | 3 |  | 8284 |  | 31.76 |  | < .001 |  | 0.350 |  |
| Target Character ✻ Group |  | 10304 |  | 3 |  | 3435 |  | 13.17 |  | < .001 |  | 0.182 |  |
| Target Character ✻ Gender |  | 2006 |  | 3 |  | 669 |  | 2.56 |  | 0.056 |  | 0.042 |  |
| Residual |  | 46166 |  | 177 |  | 261 |  |  |  |  |  |  |  |
| Note. Type 3 Sums of Squares | | | | | | | | | | | | | |
|  | | | | | | | | | | | | | |

| Between Subjects Effects | | | | | | | | | | | | | |
| --- | --- | --- | --- | --- | --- | --- | --- | --- | --- | --- | --- | --- | --- |
|  |  |  |  |  |  |  |  |  |  |  |  |  |  |
|  | | **Sum of Squares** | | **df** | | **Mean Square** | | **F** | | **p** | | **η²_p_** | |
| Group |  | 87.5 |  | 1 |  | 87.5 |  | 0.294 |  | 0.590 |  | 0.005 |  |
| Gender |  | 3196.1 |  | 1 |  | 3196.1 |  | 10.740 |  | 0.002 |  | 0.154 |  |
| Residual |  | 17556.6 |  | 59 |  | 297.6 |  |  |  |  |  |  |  |
| Note. Type 3 Sums of Squares | | | | | | | | | | | | | |
|  | | | | | | | | | | | | | |

| **3C. Perceived competence**  Within Subjects Effects | | | | | | | | | | | | | | | | | | | | | | | | | | | |
| --- | --- | --- | --- | --- | --- | --- | --- | --- | --- | --- | --- | --- | --- | --- | --- | --- | --- | --- | --- | --- | --- | --- | --- | --- | --- | --- | --- |
|  | | | |  |  | | | |  |  | | |  |  | | |  | | |  | |  | |  |  |  |  |
|  | | | | | **Sum of Squares** | | | | | **df** | | | | **Mean Square** | | | | | | **F** | | | | **p** | | **η²_p_** | |
| Target Character | | | |  | 1141 | | | |  | 3 | | |  | 380 | | |  | | | 1.162 | |  | | 0.326 |  | 0.019 |  |
| Target Character ✻ Group | | | |  | 530 | | | |  | 3 | | |  | 177 | | |  | | | 0.540 | |  | | 0.656 |  | 0.009 |  |
| Target Character ✻ Gender | | | |  | 3937 | | | |  | 3 | | |  | 1312 | | |  | | | 4.010 | |  | | 0.009 |  | 0.064 |  |
| Residual | | | |  | 57923 | | | |  | 177 | | |  | 327 | | |  | | |  | |  | |  |  |  |  |
| Note. Type 3 Sums of Squares | | | | | | | | | | | | | | | | | | | | | | | | | | | |
|  | | | | | | | | | | | | | | | | | | | | | | | | | | | |
| Between Subjects Effects | | | | | | | | | | | | | | | | | | | | | | |  |  |  |  |  |
|  |  |  |  | | |  |  |  | | |  |  | | |  |  | |  |  | |  | |  |  |  |  |  |
|  | | **Sum of Squares** | | | | **df** | | **Mean Square** | | | | **F** | | | | **p** | | | **η²_p_** | | | |  |  |  |  |  |
| Group |  | 626.1 |  | | | 1 |  | 626.1 | | |  | 1.342 | | |  | 0.251 | |  | 0.022 | |  | |  |  |  |  |  |
| Gender |  | 73.2 |  | | | 1 |  | 73.2 | | |  | 0.157 | | |  | 0.693 | |  | 0.003 | |  | |  |  |  |  |  |
| Residual |  | 27523.1 |  | | | 59 |  | 466.5 | | |  |  | | |  |  | |  |  | |  | |  |  |  |  |  |
| Note. Type 3 Sums of Squares | | | | | | | | | | | | | | | | | | | | | | |  |  |  |  |  |
|  | | | | | | | | | | | | | | | | | | | | | | |  |  |  |  |  |

**3D. Cognitive empathy**

| Within Subjects Effects | | | | | | | | | | | | | | | | | | | | | | | | | | |
| --- | --- | --- | --- | --- | --- | --- | --- | --- | --- | --- | --- | --- | --- | --- | --- | --- | --- | --- | --- | --- | --- | --- | --- | --- | --- | --- |
|  | | | | |  | |  | | |  |  |  | |  | | | |  |  | | |  |  |  |  |  |
|  | | | | | | | **Sum of Squares** | | | | **df** | | | **Mean Square** | | | | | **F** | | | | **p** | | **η²_p_** | |
| Valence | | | | |  | | 0.812 | | |  | 1 |  | | 0.812 | | | |  | 0.0158 | | |  | 0.900 |  | 0.000 |  |
| Valence ✻ Group | | | | |  | | 10.823 | | |  | 1 |  | | 10.823 | | | |  | 0.2112 | | |  | 0.648 |  | 0.004 |  |
| Valence ✻ Gender | | | | |  | | 46.977 | | |  | 1 |  | | 46.977 | | | |  | 0.9168 | | |  | 0.342 |  | 0.016 |  |
| Residual | | | | |  | | 2971.808 | | |  | 58 |  | | 51.238 | | | |  |  | | |  |  |  |  |  |
| Target Character | | | | |  | | 219.102 | | |  | 3 |  | | 73.034 | | | |  | 6.1068 | | |  | < .001 |  | 0.095 |  |
| Target Character ✻ Group | | | | |  | | 27.668 | | |  | 3 |  | | 9.223 | | | |  | 0.7712 | | |  | 0.512 |  | 0.013 |  |
| Target Character ✻ Gender | | | | |  | | 24.698 | | |  | 3 |  | | 8.233 | | | |  | 0.6884 | | |  | 0.560 |  | 0.012 |  |
| Residual | | | | |  | | 2080.957 | | |  | 174 |  | | 11.960 | | | |  |  | | |  |  |  |  |  |
| Valence ✻ Target Character | | | | |  | | 8.835 | | |  | 3 |  | | 2.945 | | | |  | 0.2542 | | |  | 0.858 |  | 0.004 |  |
| Valence ✻ Target Character ✻ Group | | | | |  | | 96.610 | | |  | 3 |  | | 32.203 | | | |  | 2.7801 | | |  | 0.043 |  | 0.046 |  |
| Valence ✻ Target Character ✻ Gender | | | | |  | | 9.155 | | |  | 3 |  | | 3.052 | | | |  | 0.2635 | | |  | 0.852 |  | 0.005 |  |
| Residual | | | | |  | | 2015.516 | | |  | 174 |  | | 11.583 | | | |  |  | | |  |  |  |  |  |
| Note. Type 3 Sums of Squares | | | | | | | | | | | | | | | | | | | | | | | | | | |
|  | | | | | | | | | | | | | | | | | | | | | | | | | | |
| Between Subjects Effects | | | | | | | | | | | | | | | | | | | | |  |  |  |  |  |  |
|  |  |  |  |  | |  | |  |  | |  | |  | |  |  |  | | |  |  |  |  |  |  |  |
|  | | **Sum of Squares** | | **df** | | | | **Mean Square** | | | **F** | | | | **p** | | **η²_p_** | | | |  |  |  |  |  |  |
| Group |  | 169 |  | 1 | |  | | 169 |  | | 0.425 | |  | | 0.517 |  | 0.007 | | |  |  |  |  |  |  |  |
| Gender |  | 741 |  | 1 | |  | | 741 |  | | 1.863 | |  | | 0.178 |  | 0.031 | | |  |  |  |  |  |  |  |
| Residual |  | 23069 |  | 58 | |  | | 398 |  | |  | |  | |  |  |  | | |  |  |  |  |  |  |  |
| Note. Type 3 Sums of Squares | | | | | | | | | | | | | | | | | | | | |  |  |  |  |  |  |
|  | | | | | | | | | | | | | | | | | | | | |  |  |  |  |  |  |

*Cognitive empathy: Sub-analyses for cocaine users*

| Within Subjects Effects | | | | | | | | | | | | | | | | | | | | | | | | | | | |
| --- | --- | --- | --- | --- | --- | --- | --- | --- | --- | --- | --- | --- | --- | --- | --- | --- | --- | --- | --- | --- | --- | --- | --- | --- | --- | --- | --- |
|  | | | | |  | |  | | |  | |  |  | |  | | | |  |  | | |  |  |  |  |  |
|  | | | | | | | **Sum of Squares** | | | | | **df** | | | **Mean Square** | | | | | **F** | | | | **p** | | **η²_p_** | |
| Valence | | | | |  | | 6.83 | | |  | | 1 |  | | 6.83 | | | |  | 0.0792 | | |  | 0.781 |  | 0.004 |  |
| Valence ✻ Gender | | | | |  | | 44.80 | | |  | | 1 |  | | 44.80 | | | |  | 0.5193 | | |  | 0.479 |  | 0.025 |  |
| Residual | | | | |  | | 1725.57 | | |  | | 20 |  | | 86.28 | | | |  |  | | |  |  |  |  |  |
| Target Character | | | | |  | | 171.45 | | |  | | 3 |  | | 57.15 | | | |  | 6.1366 | | |  | 0.001 |  | 0.235 |  |
| Target Character ✻ Gender | | | | |  | | 58.51 | | |  | | 3 |  | | 19.50 | | | |  | 2.0943 | | |  | 0.110 |  | 0.095 |  |
| Residual | | | | |  | | 558.78 | | |  | | 60 |  | | 9.31 | | | |  |  | | |  |  |  |  |  |
| Valence ✻ Target Character | | | | |  | | 42.82 | | |  | | 3 |  | | 14.27 | | | |  | 0.7430 | | |  | 0.531 |  | 0.036 |  |
| Valence ✻ Target Character ✻ Gender | | | | |  | | 23.53 | | |  | | 3 |  | | 7.84 | | | |  | 0.4083 | | |  | 0.748 |  | 0.020 |  |
| Residual | | | | |  | | 1152.52 | | |  | | 60 |  | | 19.21 | | | |  |  | | |  |  |  |  |  |
| Note. Type 3 Sums of Squares | | | | | | | | | | | | | | | | | | | | | | | | | | | |
|  | | | | | | | | | | | | | | | | | | | | | | | | | | | |
| Between Subjects Effects | | | | | | | | | | | | | | | | | | | | | |  |  |  |  |  |  |
|  |  |  |  |  | |  | |  |  | |  | | |  | |  |  |  | | |  |  |  |  |  |  |  |
|  | | **Sum of Squares** | | **df** | | | | **Mean Square** | | | **F** | | | | | **p** | | **η²_p_** | | | |  |  |  |  |  |  |
| Gender |  | 2142 |  | 1 | |  | | 2142 |  | | 7.93 | | |  | | 0.011 |  | 0.284 | | |  |  |  |  |  |  |  |
| Residual |  | 5403 |  | 20 | |  | | 270 |  | |  | | |  | |  |  |  | | |  |  |  |  |  |  |  |
| Note. Type 3 Sums of Squares | | | | | | | | | | | | | | | | | | | | | |  |  |  |  |  |  |
|  | | | | | | | | | | | | | | | | | | | | | |  |  |  |  |  |  |

*Cognitive empathy: Sub-analyses for control participants*

| Within Subjects Effects | | | | | | | | | | | | | |
| --- | --- | --- | --- | --- | --- | --- | --- | --- | --- | --- | --- | --- | --- |
|  |  |  |  |  |  |  |  |  |  |  |  |  |  |
|  | | **Sum of Squares** | | **df** | | **Mean Square** | | **F** | | **p** | | **η²_p_** | |
| Valence |  | 14.8 |  | 1 |  | 14.76 |  | 0.442 |  | 0.510 |  | 0.012 |  |
| Valence ✻ Gender |  | 14.0 |  | 1 |  | 13.96 |  | 0.419 |  | 0.522 |  | 0.011 |  |
| Residual |  | 1234.5 |  | 37 |  | 33.36 |  |  |  |  |  |  |  |
| Target Character |  | 127.2 |  | 3 |  | 42.40 |  | 3.232 |  | 0.025 |  | 0.080 |  |
| Target Character ✻ Gender |  | 32.2 |  | 3 |  | 10.74 |  | 0.819 |  | 0.486 |  | 0.022 |  |
| Residual |  | 1456.1 |  | 111 |  | 13.12 |  |  |  |  |  |  |  |
| Valence ✻ Target Character |  | 79.4 |  | 3 |  | 26.45 |  | 3.594 |  | 0.016 |  | 0.089 |  |
| Valence ✻ Target Character ✻ Gender |  | 31.5 |  | 3 |  | 10.51 |  | 1.427 |  | 0.239 |  | 0.037 |  |
| Residual |  | 817.1 |  | 111 |  | 7.36 |  |  |  |  |  |  |  |
| Note. Type 3 Sums of Squares | | | | | | | | | | | | | |
|  | | | | | | | | | | | | | |

| Between Subjects Effects | | | | | | | | | | | | | |
| --- | --- | --- | --- | --- | --- | --- | --- | --- | --- | --- | --- | --- | --- |
|  |  |  |  |  |  |  |  |  |  |  |  |  |  |
|  | | **Sum of Squares** | | **df** | | **Mean Square** | | **F** | | **p** | | **η²_p_** | |
| Gender |  | 2.09 |  | 1 |  | 2.09 |  | 0.00476 |  | 0.945 |  | 0.000 |  |
| Residual |  | 16263.03 |  | 37 |  | 439.54 |  |  |  |  |  |  |  |
| Note. Type 3 Sums of Squares | | | | | | | | | | | | | |
|  | | | | | | | | | | | | | |

**3E. Emotional empathy**

| Within Subjects Effects | | | | | | | | | | | | | |
| --- | --- | --- | --- | --- | --- | --- | --- | --- | --- | --- | --- | --- | --- |
|  |  |  |  |  |  |  |  |  |  |  |  |  |  |
|  | | **Sum of Squares** | | **df** | | **Mean Square** | | **F** | | **p** | | **η²_p_** | |
| Valence |  | 110.049 |  | 1 |  | 110.049 |  | 1.05770 |  | 0.308 |  | 0.018 |  |
| Valence ✻ Group |  | 0.376 |  | 1 |  | 0.376 |  | 0.00361 |  | 0.952 |  | 0.000 |  |
| Valence ✻ Gender |  | 180.249 |  | 1 |  | 180.249 |  | 1.73241 |  | 0.193 |  | 0.029 |  |
| Residual |  | 6034.635 |  | 58 |  | 104.045 |  |  |  |  |  |  |  |
| Target Character |  | 819.215 |  | 3 |  | 273.072 |  | 5.06850 |  | 0.002 |  | 0.080 |  |
| Target Character ✻ Group |  | 471.474 |  | 3 |  | 157.158 |  | 2.91702 |  | 0.036 |  | 0.048 |  |
| Target Character ✻ Gender |  | 267.914 |  | 3 |  | 89.305 |  | 1.65759 |  | 0.178 |  | 0.028 |  |
| Residual |  | 9374.452 |  | 174 |  | 53.876 |  |  |  |  |  |  |  |
| Valence ✻ Target Character |  | 25.844 |  | 3 |  | 8.615 |  | 0.51775 |  | 0.671 |  | 0.009 |  |
| Valence ✻ Target Character ✻ Group |  | 28.189 |  | 3 |  | 9.396 |  | 0.56473 |  | 0.639 |  | 0.010 |  |
| Valence ✻ Target Character ✻ Gender |  | 7.187 |  | 3 |  | 2.396 |  | 0.14397 |  | 0.933 |  | 0.002 |  |
| Residual |  | 2895.156 |  | 174 |  | 16.639 |  |  |  |  |  |  |  |
| Note. Type 3 Sums of Squares | | | | | | | | | | | | | |
|  | | | | | | | | | | | | | |

| Between Subjects Effects | | | | | | | | | | | | | |
| --- | --- | --- | --- | --- | --- | --- | --- | --- | --- | --- | --- | --- | --- |
|  |  |  |  |  |  |  |  |  |  |  |  |  |  |
|  | | **Sum of Squares** | | **df** | | **Mean Square** | | **F** | | **p** | | **η²_p_** | |
| Group |  | 3.12 |  | 1 |  | 3.12 |  | 0.00389 |  | 0.950 |  | 0.000 |  |
| Gender |  | 1765.99 |  | 1 |  | 1765.99 |  | 2.20197 |  | 0.143 |  | 0.037 |  |
| Residual |  | 46516.41 |  | 58 |  | 802.01 |  |  |  |  |  |  |  |
| Note. Type 3 Sums of Squares | | | | | | | | | | | | | |
|  | | | | | | | | | | | | | |

**3F. Self-related expectancies**

| Within Subjects Effects | | | | | | | | | | | | | | | | | | | | | | | | | |
| --- | --- | --- | --- | --- | --- | --- | --- | --- | --- | --- | --- | --- | --- | --- | --- | --- | --- | --- | --- | --- | --- | --- | --- | --- | --- |
|  | | | |  |  | | | |  |  | |  | |  | | |  | |  | |  |  |  |  |  |
|  | | | | | **Sum of Squares** | | | | | **df** | | | | **Mean Square** | | | | | **F** | | | **p** | | **η²_p_** | |
| Scenario Valence | | | |  | 6740.1 | | | |  | 1 | |  | | 6740.1 | | |  | | 69.118 | |  | < .001 |  | 0.531 |  |
| Scenario Valence ✻ Group | | | |  | 127.4 | | | |  | 1 | |  | | 127.4 | | |  | | 1.307 | |  | 0.257 |  | 0.021 |  |
| Scenario Valence ✻ Gender | | | |  | 28.6 | | | |  | 1 | |  | | 28.6 | | |  | | 0.294 | |  | 0.590 |  | 0.005 |  |
| Residual | | | |  | 5948.5 | | | |  | 61 | |  | | 97.5 | | |  | |  | |  |  |  |  |  |
| Note. Type 3 Sums of Squares | | | | | | | | | | | | | | | | | | | | | | | | | |
|  | | | | | | | | | | | | | | | | | | | | | | | | | |
| Between Subjects Effects | | | | | | | | | | | | | | | | | | | | |  |  |  |  |  |
|  |  |  |  | | |  |  |  | | |  | |  | |  |  |  |  | |  |  |  |  |  |  |
|  | | **Sum of Squares** | | | | **df** | | **Mean Square** | | | | | **F** | | | **p** | | **η²_p_** | | |  |  |  |  |  |
| Group |  | 1360 |  | | | 1 |  | 1360 | | |  | | 6.66 | |  | 0.012 |  | 0.098 | |  |  |  |  |  |  |
| Gender |  | 754 |  | | | 1 |  | 754 | | |  | | 3.69 | |  | 0.059 |  | 0.057 | |  |  |  |  |  |  |
| Residual |  | 12461 |  | | | 61 |  | 204 | | |  | |  | |  |  |  |  | |  |  |  |  |  |  |
| Note. Type 3 Sums of Squares | | | | | | | | | | | | | | | | | | | | |  |  |  |  |  |
|  | | | | | | | | | | | | | | | | | | | | |  |  |  |  |  |
